# SLAM Project - Long Term Ecological Study of the Impacts of Climate Change in the Natural Forest of Azores: II - A survey of exotic arthropods in disturbed forest habitats

**DOI:** 10.3897/BDJ.10.e81410

**Published:** 2022-03-29

**Authors:** Paulo A. V. Borges, Lucas Lamelas-Lopez, Peter E. Stüben, Alejandra Ros-Prieto, Rosalina Gabriel, Mário Boieiro, Noelline Tsafack, Maria Teresa Ferreira

**Affiliations:** 1 cE3c—Centre for Ecology, Evolution and Environmental Changes/Azorean Biodiversity Group, Faculty of Agriculture and Environment, Universidade dos Açores, 9700-042, Angra do Heroísmo, Azores, Portugal cE3c—Centre for Ecology, Evolution and Environmental Changes/Azorean Biodiversity Group, Faculty of Agriculture and Environment, Universidade dos Açores, 9700-042 Angra do Heroísmo, Azores Portugal; 2 IUCN SSC Mid-Atlantic Islands Invertebrates Specialist Group, Angra do Heroísmo, Azores, Portugal IUCN SSC Mid-Atlantic Islands Invertebrates Specialist Group Angra do Heroísmo, Azores Portugal; 3 CHANGE – Global Change and Sustainability Institute, Angra do Heroísmo, Azores, Portugal CHANGE – Global Change and Sustainability Institute Angra do Heroísmo, Azores Portugal; 4 CURCULIO Institute, Hauweg 62, D-41066, Mönchengladbach, Germany CURCULIO Institute, Hauweg 62, D-41066 Mönchengladbach Germany; 5 Regional Secretariat of Environment and Climate Change, Project LIFE BEETLES (LIFE 18 NAT/PT/000864), Rua do Galo n118, 9700-040, Angra do Heroísmo, Azores, Portugal Regional Secretariat of Environment and Climate Change, Project LIFE BEETLES (LIFE 18 NAT/PT/000864), Rua do Galo n118, 9700-040 Angra do Heroísmo, Azores Portugal

**Keywords:** Arthropoda, Azores, endemic species, exotic species, exotic forest, inventory, Macaronesia, long-term sampling, SLAM traps

## Abstract

**Background:**

The data we present consist of an inventory of exotic arthropods, potentially invasive, collected in exotic and mixed forests and disturbed native forest patches of the Azores Archipelago. The study was carried out between 2019 and 2020 in four islands: Corvo, Flores, Terceira and Santa Maria, where a total of 45 passive flight interception SLAM traps were deployed, during three to six consecutive months. This manuscript is the second contribution of the “SLAM Project - Long Term Ecological Study of the Impacts of Climate Change in the Natural Forest of Azores”.

**New information:**

We provide an inventory of terrestrial arthropods belonging to Arachnida, Diplopoda, Chilopoda and Insecta classes from four Azorean islands. We identified a total of 21,175 specimens, belonging to 20 orders, 93 families and 249 species of arthropods. A total of 125 species are considered introduced, 89 native non-endemic and 35 endemic. We registered 34 new records (nine for Corvo, three for Flores, six for Terceira and 16 for Santa Maria), of which five are new for Azores, being all exotic possibly recently introduced: *Dieckmanniellusnitidulus* (Gyllenhal, 1838), *Gronopsfasciatus* Küster, 1851, *Hadroplontustrimaculatus* (Fabricius, 1775), *Hypurusbertrandi* (Perris, 1852) (all Coleoptera, Curculionidae) and *Cardiocondylamauritanica* Forel, 1890 (Hymenoptera, Formicidae). This publication highlights the importance of planted forests and disturbed native forest patches as reservoirs of potentially invasive arthropods and refuges for some rare relict endemic arthropod species.

## Introduction

Arthropod communities, particularly insects, are being affected by unprecedented and rapid population declines (Hallmann et al. 2017, Sánchez-Bayo and Wyckhuys 2019, Cardoso et al. 2020, [Bibr B7659697][Bibr B7659896]). The most important causes for this biodiversity loss are habitat loss, degradation and fragmentation, climate change and the introduction and spread of invasive species (Russell et al. 2017, Borges et al. 2019a). In this context, the biodiversity of oceanic islands has been especially and dramatically affected by these drivers as consequence of human colonisation, global trade and tourism (Triantis et al. 2010, Borges et al. 2019b, Cowie et al. 2022, Stüben 2022).

In the case of Azores islands, since Portuguese settlement in the 15^th^ century, the original landscape was strongly altered by replacing pristine and native forest areas with exotic tree plantations, crops, pastures and urban areas (Triantis et al. 2010, Borges et al. 2019b, Norder et al. 2020). Currently, the remaining native forest covers only about 5% of the total surface of the Archipelago, being restricted to the higher elevation and inaccessible areas of the islands (Gaspar et al. 2008, Triantis et al. 2010, Stüben and Borges 2019, Norder et al. 2020).

Native forest destruction (Triantis et al. 2010) and the consequent lack of connectivity between forest patches (Aparício et al. 2018), climate change (Ferreira et al. 2016) and invasive species are the main factors that contribute to arthropod decline in Azores (Stüben 2003, Stüben 2004, Borges et al. 2019b). Previous studies demonstrated that endemic species of Azorean arthropods are restricted mainly to native vegetation dominated habitats, while introduced species usually occupy human-altered habitats (Cardoso et al. 2009, Florencio et al. 2015, Florencio et al. 2016). Additionally, the proportion of introduced arthropod species in Azores is higher than native (around 60%) and, due to the higher adaptability to environmental conditions of many introduced species, they represent one of the main threats to indigenous biota in the native forest areas (Borges et al. 2019b). Moreover, Tsafack et al. (2021) showed the importance of isolated and small native forest patches, as well exotic and mixed forests close to native areas, which can function as refuges for native and rare endemic species, playing a relevant role for conservation of native biota outside Azorean protected areas.

This publication is the second data paper of the project “SLAM Project - Long Term Ecological Study of the Impacts of Climate Change in the Natural Forest of Azores” (see first in Costa and Borges 2021) that aims to monitor the distribution and abundance of arthropods in native forests from Azores using SLAM traps (Sea, Land and Air Malaise traps). Additional publications, using data coming from this project, tested specific ecological questions, namely patterns of seasonal variation on species abundance (Borges et al. 2017), patterns of temporal beta diversity in native and exotic species (Matthews et al. 2018), the potential decline of endemic insects (Borges et al. 2020), patterns of arthropod diversity in Azorean urban gardens (Arteaga et al. 2020), patterns of species richness and beta diversity in a small elevational gradient (de Vries et al. 2021) and the investigation of the role of small lowland patches of exotic forests as refuges for rare endemic Azorean arthropods (Tsafack et al. 2021).

In this second data paper, we aim to: i) survey arthropods in exotic and mixed forests and small disturbed remnants of native forests; ii) investigate the occurrence and current distribution of exotic (potentially invasive) arthropods in those habitats; and also iii) investigate the occurrence of rare endemic arthropods in those habitats.

## General description

### Purpose

This publication provides an inventory of arthropods present in exotic and mixed forests of four Azores Islands (Corvo, Flores, Terceira and Santa Maria), as well as from small remnants of disturbed native forests in three Islands (Flores, Terceira and Santa Maria).

### Additional information

The data we present are part of the long-term project SLAM (Long Term Ecological Study of the Impacts of Climate Change in the Natural Forest of Azores) that started in 2012 aiming to understand the impact of biodiversity erosion drivers on Azorean native forests (Azores, Macaronesia, Portugal). Passive flight interception SLAM traps (Sea, Land and Air Malaise traps) are being used to sample native forest plots in several Azorean islands (Costa and Borges 2021).

## Project description

### Title

SLAM Project II - A survey of exotic and endemic arthropods in Azorean disturbed Azorean forest habitats

### Personnel

The project was conceived and led by Paulo A.V. Borges.

Fieldwork: Corvo Island - Alejandra Ros-Prieto, Maria Teresa Ferreira, Mário Boieiro, Paulo A. V. Borges, Rosalina Gabriel; Flores Island - Alejandra Ros-Prieto, Maria Teresa Ferreira, Mário Boieiro, Paulo A. V. Borges, Rosalina Gabriel; Terceira Island - Alejandra Ros-Prieto, Paulo A. V. Borges, Rosalina Gabriel; Santa Maria Island - Alejandra Ros-Prieto, Nelson Moura, Paulo A. V. Borges, Rosalina Gabriel.

Parataxonomists: Alejandra Ros-Prieto, Jonne Bonnet and Sébastien Lhoumeau.

Taxonomists: Paulo A. V. Borges, Mário Boieiro and Peter E. Stüben.

Voucher specimen management was mainly undertaken by Alejandra Ros-Prieto and Paulo A. V. Borges.

### Study area description

**Figure 1.**
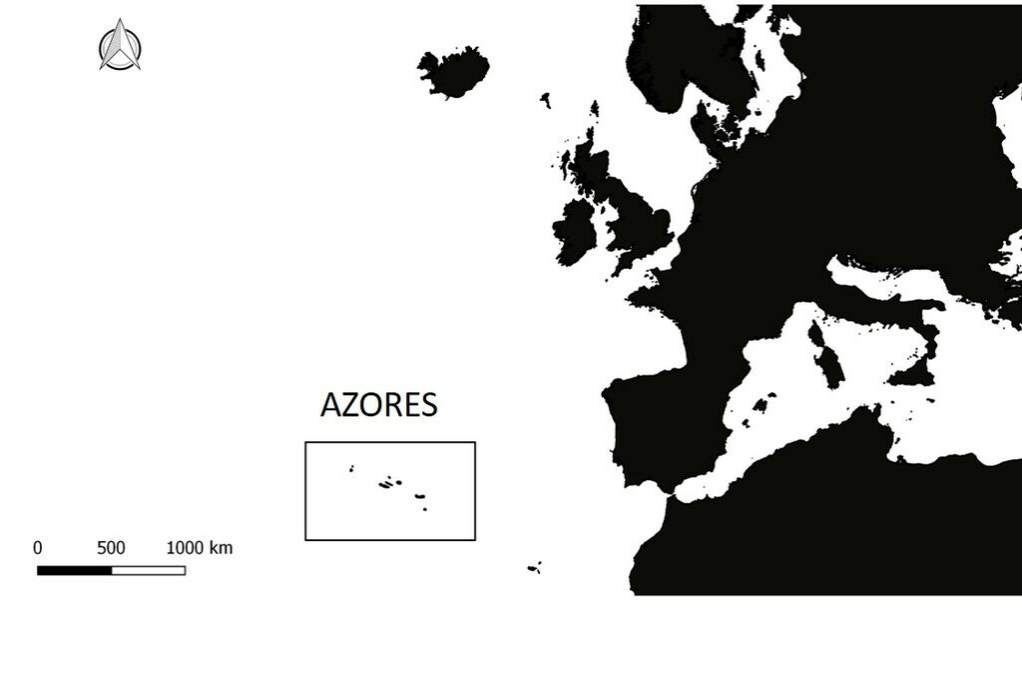
The Azores Archipelago location (Credit: Enésima Pereira, Azorean Biodiversity Group).

The study area comprises Corvo, Flores, Terceira and Santa Maria Islands, in the Azores Archipelago, located in the North Atlantic, roughly at 38°43'21"N 27°13'14"W and 38°27'30"N 28°19'22"W (Fig. 1). The climate is temperate oceanic, with regular and abundant rainfall, high levels of relative humidity and persistent winds, mainly during the winter and autumn seasons. The exotic forests are located at lower and mid-elevations and are dominated mainly by *Pittosporumundulatum* Vent., *Eucalyptus* spp., *Cryptomeriajaponica* D.Don, *Acaciamelanoxylon* R.Br. and *Pinuspinaster* Aiton. The studied native forests are located at several elevations and are mainly dominated by *Ericaazorica* Hochst. ex Seub., *Laurusazorica* (Seub.) Franco, *Ilexazorica* Gand. and *Juniperusbrevifolia* (Hochst. ex Seub.). Mixed forests included both exotic and native tree species.

### Design description

**Figure 2.**
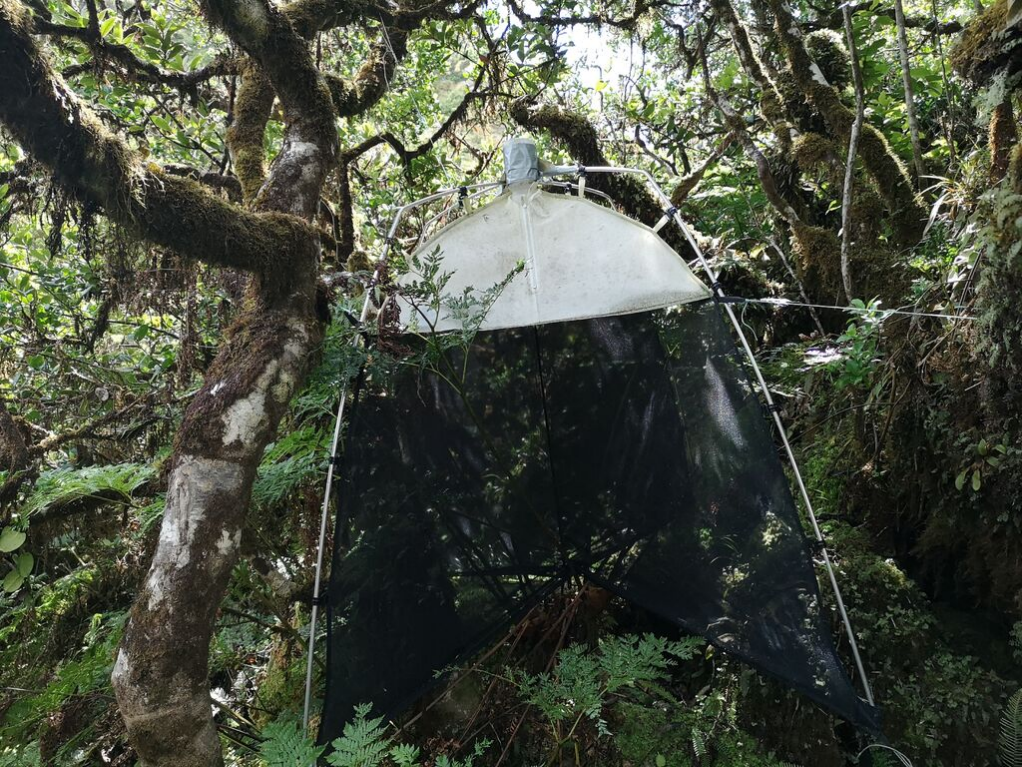
Passive flight interception SLAM trap (Sea, Land and Air Malaise traps) (Credit: Paulo A. V. Borges).

Passive flight interception SLAM traps (Sea, Land and Air Malaise traps) (Fig. 2) were used to sample 45 sites in the four study Islands (Corvo (n = 1), Flores (n = 5), Santa Maria (n =16) and Terceira (n = 23)) with one trap being set up at each plot. Although this protocol was originally developed to sample flying arthropods, by working as an extension of the tree, non-flying species can also crawl into the trap (Borges et al. 2017), enhancing the range of groups that can be sampled by this technique. Recent studies have used this sampling technique to study diversity and abundance variations in the communities of arthropod on Azorean native areas (Borges et al. 2017, Matthews et al. 2018, Borges et al. 2020, de Vries et al. 2021, Tsafack et al. 2021). The samples were collected every three or six months depending on sites. The collected specimens were sorted to morphospecies and posteriorly identified at species level by an expert taxonomist in laboratory.

### Funding

Portuguese National Funds, through FCT – Fundação para a Ciência e a Tecnologia, within the project UID/BIA/00329/2013-2023.

Direcção Regional do Ambiente - PRIBES (LIFE17 IPE/PT/000010) (2019-2020).

Direcção Regional do Ambiente – LIFE-BETTLES (LIFE18 NAT_PT_000864) (2020-2024).

AZORESBIOPORTAL –PORBIOTA (ACORES-01-0145-FEDER-000072) (2019-2022).

The database management and Open Access was funded by the project “MACRISK-Trait-based prediction of extinction risk and invasiveness for Northern Macaronesian arthropods” Fundação para a Ciência e Tecnologia FCT - PTDC/BIA-CBI/0625/2021 (2022-2024).

## Sampling methods

### Study extent

The study was conducted in four Islands of the Azores Archipelago, Corvo, Flores, Terceira and Santa Maria. The sampled habitats included exotic, mixed and disturbed native forest patches (Table 1).

### Sampling description

**Figure 3.**
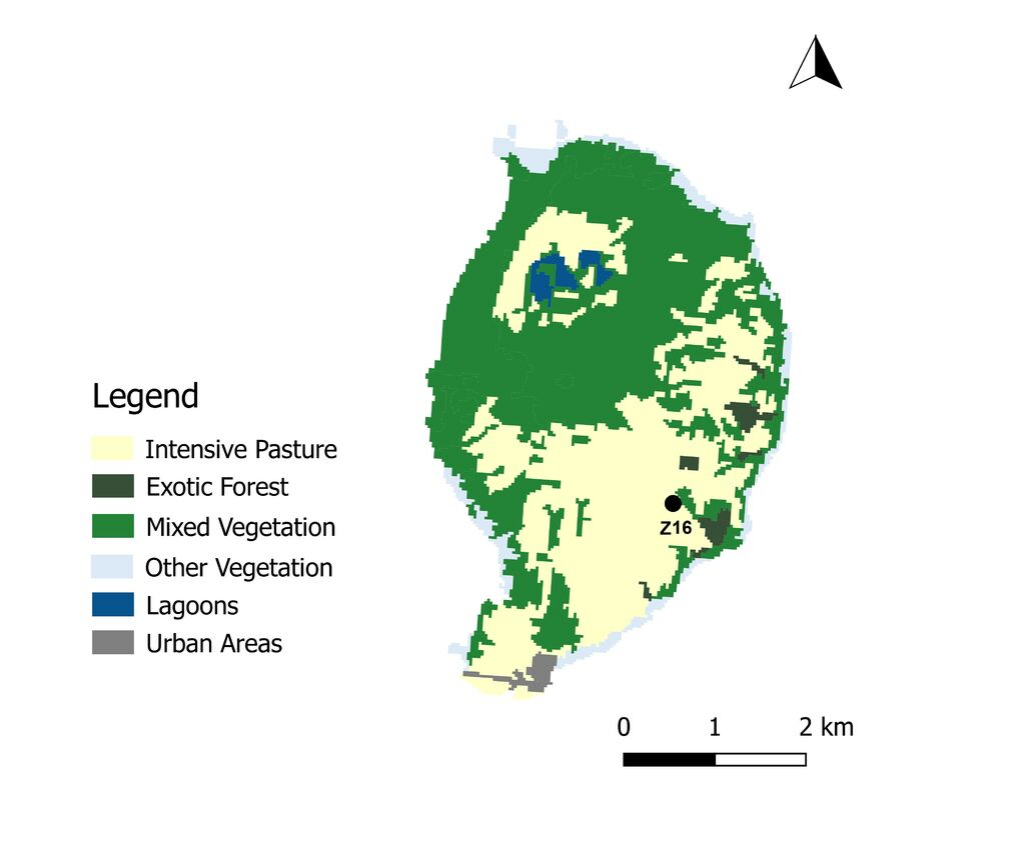
Map with the location of the sampling sites in Corvo Island, Azores. Codes of sites as in Table 1 (Land-use data extracted from Cruz et al. 2007) (Credit: Enésima Pereira, Azorean Biodiversity Group).

**Figure 4.**
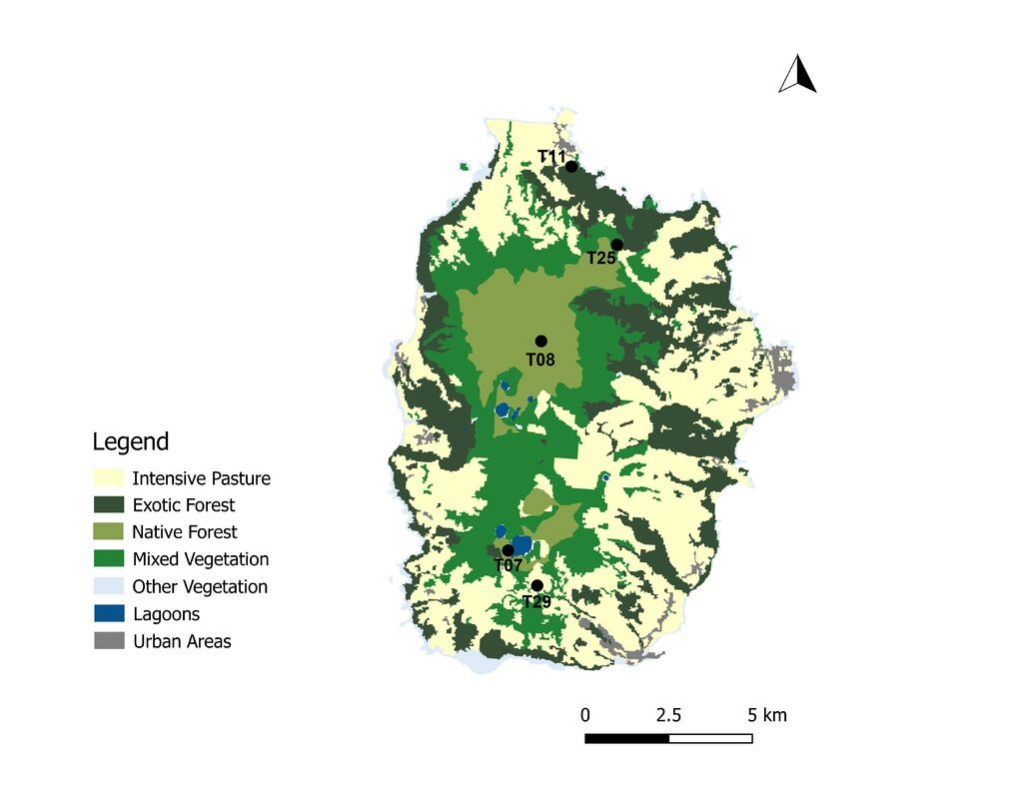
Map with the location of the sampling sites in Flores Island, Azores. Codes of sites as in Table 1 (Land-use data extracted from Cruz et al. 2007) (Credit: Enésima Pereira, Azorean Biodiversity Group).

**Figure 5.**
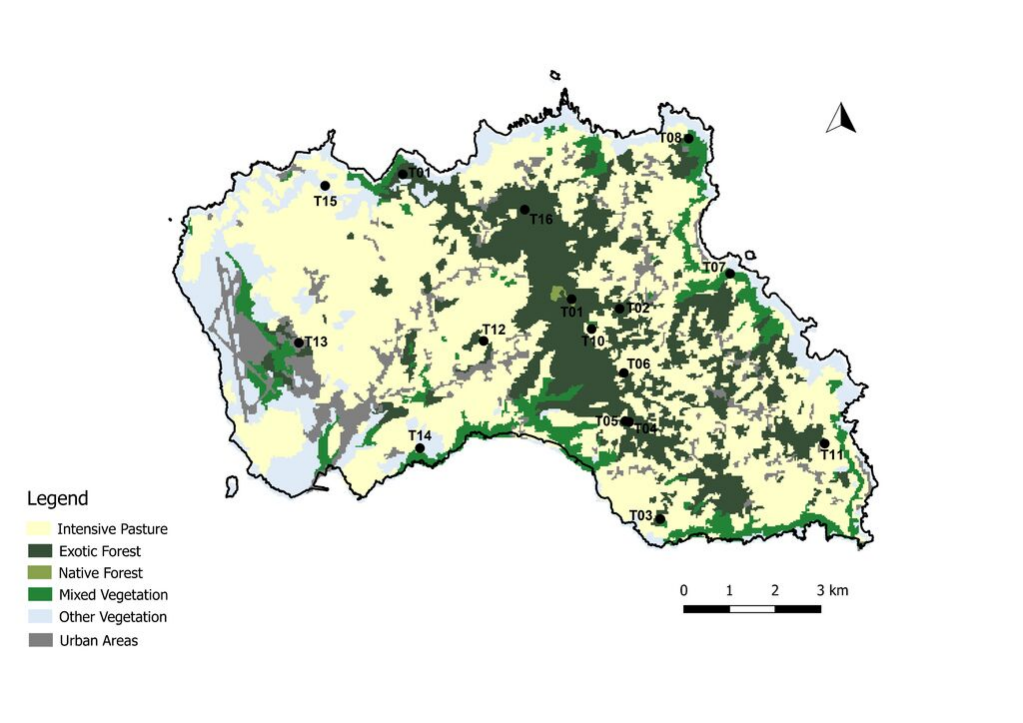
Map with the location of the sampling sites in Santa Maria Island, Azores. Codes of sites as in Table 1 (Land-use data extracted from Cruz et al. 2007) (Credit: Enésima Pereira, Azorean Biodiversity Group).

**Figure 6.**
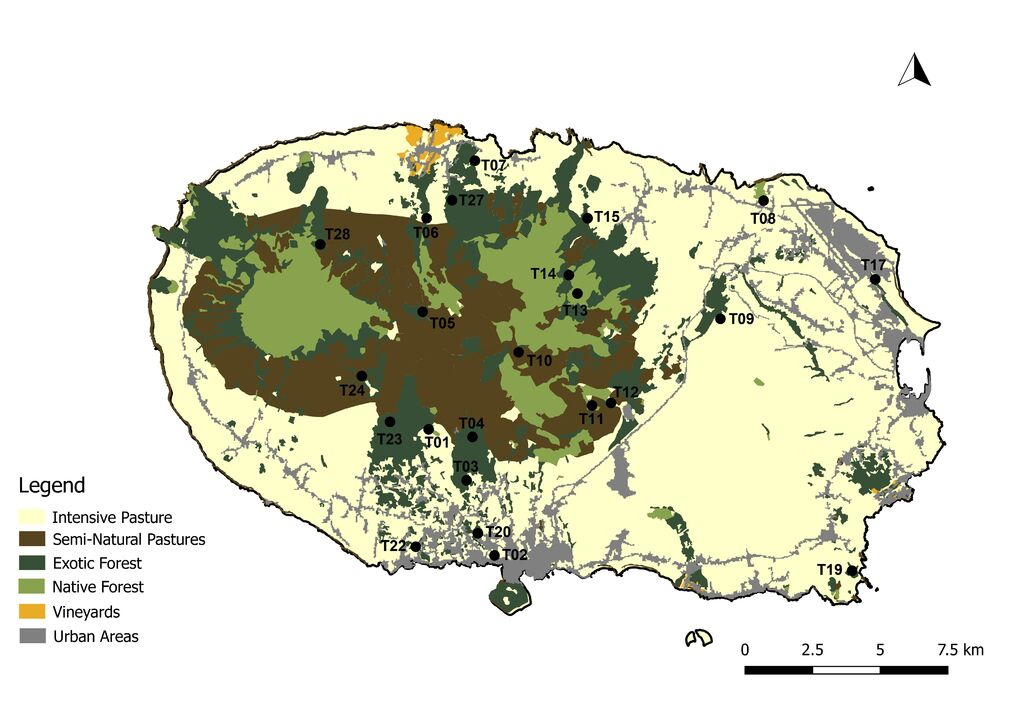
Map with the location of the sampling sites in Terceira Island, Azores. Codes of sites as in Table 1 (Land-use data extracted from Cruz et al. 2007) (Credit: Enésima Pereira, Azorean Biodiversity Group).

A total of 45 passive flight interception SLAM traps (Sea, Land and Air Malaise traps) were used to sample the plots in the four study Islands, with one trap being set up at each plot. Trap size is of approximately 110 x 110 x 110 cm. The trap functions on the basis of intercepting arthropods that crawl up the mesh and then fall inside the sampling recipient, which is filled with propylene glycol (pure 1,2-propanodiol) (Borges et al. 2017). A total of 19 SLAM traps were deployed in exotic forest areas, eight on native forest patches and 18 on mixed forests. The trap samples were collected every three months in Flores and Corvo and six months in Terceira and Santa Maria. In Corvo Island, one trap was available in a mixed forest (Fig. 3; Table 1). In Flores Island, five traps were available in both exotic forests and native forests (Fig. 4; Table 1). In Santa Maria Island, a total of 16 traps were available with only three located in disturbed native forest patches (Fig. 5; Table 1). Finally, in Terceira Island, 23 traps were available with only four in disturbed native forest patches (Fig. 6; Table 1).

### Quality control

All sampled individuals were first sorted by trained paratoxonomists (see list above). All specimens were allocated to a taxonomic species by Paulo A. V. Borges. Despite the uncertainty of juvenile identification, juveniles are also included in the data presented in this paper, since the low diversity allowed a relatively precise identification of this life-stage in Azores.

### Step description

At the laboratory, specimen sorting and arthropod identification followed standard procedures during the last 20 years or arthropod surveys in Azores. First, a combination of morphological and anatomical characters and reproductive structures was used for morphospecies creation. After, morphospecies were sent to experts for proper identification. With this procedure, a reference collection was made for all collected specimens by assigning them a morphospecies code number and respective taxonomic name and depositing them at the Dalberto Teixeira Pombo Insect Collection, University of Azores. Colonisation status of the species was obtained from the last updated checklist of Azorean arthropods (Borges et al. 2010).

## Geographic coverage

### Description

Corvo, Flores, Terceira and Santa Maria Islands, in the Azores Archipelago (Portugal).

### Coordinates

36.90597988519294 and 39.740986355883564 Latitude; -31.2945556640625 and -24.949951171875 Longitude.

## Taxonomic coverage

### Description

The following Classes and Orders are covered:

Arachnida: Araneae; Opiliones; Pseudoscorpiones

Chilopoda: Geophilomorpha; Lithobiomorpha; Scolopendromorpha; Scutigeromorpha

Diplopoda: Julida; Polydesmida

Insecta: Archaeognatha; Blattodea; Coleoptera; Dermaptera; Hemiptera; Hymenoptera; Isoptera; Neuroptera; Orthoptera; Phasmatodea; Psocoptera; Thysanoptera; Trichoptera.

### Taxa included

**Table:** 

Rank	Scientific Name	Common Name
order	Araneae	Spiders
order	Opiliones	Harvestmen
order	Pseudoscorpiones	Pseudoscorpions
class	Chilopoda	Centipedes
class	Diplopoda	Millipedes
order	Archaeognatha	Bristletails
order	Blattodea	Cockroaches
order	Coleoptera	Beetles
order	Dermaptera	Earwig
order	Hemiptera	Bugs
order	Hymenoptera	Ants
order	Isoptera	Termites
order	Neuroptera	Lacewings
order	Orthoptera	Grasshoppers, crickets
order	Phasmatodea	Stick insects
order	Psocodea	Booklice
order	Thysanoptera	Thrips
order	Trichoptera	Caddisflies

## Collection data

### Collection name

Entomoteca Dalberto Teixeira Pombo (DTP); University of Azores

### Collection identifier

DTP

### Specimen preservation method

All specimens were preserved in 96% ethanol.

### Curatorial unit

Curator: Paulo A. V. Borges

## Usage licence

### Usage licence

Creative Commons Public Domain Waiver (CC-Zero)

## Data resources

### Data package title

A survey of exotic arthropods in disturbed Azorean forest habitats using SLAM traps.

### Resource link

http://ipt.gbif.pt/ipt/resource?r=pribes_exotic_arthropods

### Alternative identifiers

https://www.gbif.org/dataset/020231d8-39b6-478f-ac24-715bf97c8ef4

### Number of data sets

2

### Data set 1.

#### Data set name

Event Table

#### Data format

Darwin Core Archive format

#### Number of columns

24

#### Character set

UTF-8

#### Download URL

http://ipt.gbif.pt/ipt/resource?r=pribes_exotic_arthropods

#### Data format version

version 1.5

#### Description

The dataset was published in Global Biodiversity Information Facility platform, GBIF (Borges et al. 2022). The following data table includes all the records for which a taxonomic identification of the species was possible. The dataset submitted to GBIF is structured as a sample event dataset that has been published as a Darwin Core Archive (DwCA), which is a standardised format for sharing biodiversity data as a set of one or more data tables. The core data file contains 45 records (eventID). This IPT (Integrated Publishing Toolkit) archives the data and thus serves as the data repository. The data and resource metadata are available for download in the Portuguese GBIF Portal IPT (Borges et al. 2022).

**Data set 1.** 

Column label	Column description
id	Unique identification code for sampling event data.
eventID	Identifier of the events, unique for the dataset.
samplingProtocol	The sampling protocol used to capture the species.
sampleSizeValue	The numeric amount of time spent in each sampling.
sampleSizeUnit	The unit of the sample size value.
eventDate	Date or date range the record was collected.
year	Year of the event.
minimumElevationInMetres	The lower limit of the range of elevation (altitude, usually above sea level), in metres.
verbatimEventDate	The verbatim original representation of the date and time information for an Event. In this case, we use the season and year.
habitat	The habitat of the sample.
locationID	Identifier of the location.
islandGroup	Name of archipelago.
island	Name of the island.
country	Country of the sampling site.
countryCode	ISO code of the country of the sampling site.
stateProvince	Name of the region of the sampling site.
municipality	Municipality of the sampling site.
locality	Name of the locality.
decimalLatitude	Approximate centre point decimal latitude of the field site in GPS coordinates.
decimalLongitude	Approximate centre point decimal longitude of the field site in GPS coordinates.
geodeticDatum	The ellipsoid, geodetic datum or spatial reference system (SRS) upon which the geographic coordinates given in decimalLatitude and decimalLongitude are based.
coordinateUncertaintyInMetres	Uncertainty of the coordinates of the centre of the sampling plot in metres.
coordinatePrecision	A decimal representation of the precision of the coordinates given in the decimalLatitude and decimalLongitude.
georeferenceSources	A list (concatenated and separated) of maps, gazetteers or other resources used to georeference the Location, described specifically enough to allow anyone in the future to use the same resources.

### Data set 2.

#### Data set name

Occurrence Table

#### Data format

Darwin Core Archive format

#### Number of columns

30

#### Character set

UTF-8

#### Download URL

https://www.gbif.org/dataset/020231d8-39b6-478f-ac24-715bf97c8ef4

#### Data format version

version 1.5

#### Description

The dataset was published in Global Biodiversity Information Facility platform, GBIF (Borges et al. 2022). The following data table includes all the records for which a taxonomic identification of the species was possible. The dataset submitted to GBIF is structured as an occurrence table that has been published as a Darwin Core Archive (DwCA), which is a standardised format for sharing biodiversity data as a set of one or more data tables. The core data file contains 2095 records (occurrenceID). This IPT (Integrated Publishing Toolkit) archives the data and thus serves as the data repository. The data and resource metadata are available for download in the Portuguese GBIF Portal IPT (Borges et al. 2022).

**Data set 2.** 

Column label	Column description
id	Unique identification code for sampling event data.
type	Type of the record, as defined by the Public Core standard.
licence	Reference to the licence under which the record is published.
institutionID	The identity of the institution publishing the data.
institutionCode	The code of the institution publishing the data.
collectionID	The identity of the collection publishing the data.
collectionCode	The code of the collection where the specimens are conserved.
basisOfRecord	The nature of the data record.
occurrenceID	Identifier of the record, coded as a global unique identifier.
recordedBy	A list (concatenated and separated) of names of people, groups or organisations who performed the sampling in the field.
organismQuantity	A number or enumeration value for the quantity of organisms.
organismQuantityType	The type of quantification system used for the quantity of organisms.
sex	The sex and quantity of the individuals captured.
lifeStage	The life stage of the organisms captured.
establishmentMeans	The process of establishment of the species in the location, using a controlled vocabulary: in the GBIF database, we used the Borges et al. (2010) original data: 'native', 'introduced', 'endemic'.
eventID	Identifier of the events, unique for the dataset.
identifiedBy	A list (concatenated and separated) of names of people, groups or organisations who assigned the Taxon to the subject.
dateIdentified	The date on which the subject was determined as representing the Taxon.
identificationRemarks	Information about morphospecies identification (code in Dalberto Teixeira Pombo Collection).
scientificName	Complete scientific name including author and year.
kingdom	Kingdom name.
phylum	Phylum name.
class	Class name.
order	Order name.
family	Family name.
genus	Genus name.
specificEpithet	Specific epithet.
infraspecificEpithet	Infrapecific epithet.
taxonRank	Lowest taxonomic rank of the record.
scientificNameAuthorship	Name of the author of the lowest taxon rank included in the record.

## Additional information

We collected a total of 27,958 specimens (Suppl. material 1; Borges et al. 2022) from which it was possible to identify to species level 76% of the specimens (21,175) (Table 2). These identified specimens belong to 20 orders, 93 families and 249 species of arthropods. A total of 125 species are considered introduced, 89 native non-endemic and 35 endemic (Table 2). Additionally, a total of 147 taxa were recorded at genus, family or order level (Suppl. material 1).

Just considering the 249 arthropod species identified at archipelago level, the five most abundant species were the native bug *Cyphopterumadcendens* (Herrich-Schäffer, 1835) (n = 1707), the ants *Lasiusgrandis* Forel, 1909 (n = 1273) and *Monomoriumcarbonarium* (F. Smith, 1858) (n = 1096), the endemic cixiid *Cixiusazoterceirae* Remane & Asche, 1979 (n = 968) and the native harvestmen *Leiobunumblackwalli* Meade, 1861 (n = 873) (Table 2).

At island scale, the native ant *Lasiusgrandis* was also one of the most abundant arthropods in Corvo (n = 31) and Santa Maria (n = 348). Curiously, in both Islands, one of the two most abundant species represent a new island record, being the exotic spider *Porrhoclubionagenevensis* (L. Koch, 1866) (n = 15), new for Corvo and the exotic (possibly invasive) beetle *Lagriahirta* (Linnaeus, 1758) (n =382), new for Santa Maria.

In Flores, the native *Trichopsocusclarus* (Banks, 1908) (n = 143) and the endemic *Eupteryxazorica* Ribaut, 1941 (n = 23) were the most abundant arthropod species. Finally, in Terceira, the native and endemic Hemiptera, respectively *Cyphopterumadcendens* (n = 1613) and *Cixiusazoterceirae* (n = 968), were the most abundant species (Table 2).

### New Azores species records

In this study, we registered a total of 34 new records for one or more islands of Azores (nine for Corvo, three for Flores, six for Terceira and 16 for Santa Maria), of which the curculionids *Dieckmanniellusnitidulus* (Gyllenhal, 1838), *Gronopsfasciatus* Küster, 1851, *Hadroplontustrimaculatus* (Fabricius, 1775) and *Hypurusbertrandi* (Perris, 1852) are new records for Azores. In addition, the ant *Cardiocondylamauritanica* Forel, 1890 (Hymenoptera, Formicidae) is also a new record for Azores. All these species are exotics, possibly recently introduced.

*Dieckmanniellusnitidulus* (Gyllenhal, 1838)

**Figure 7.**
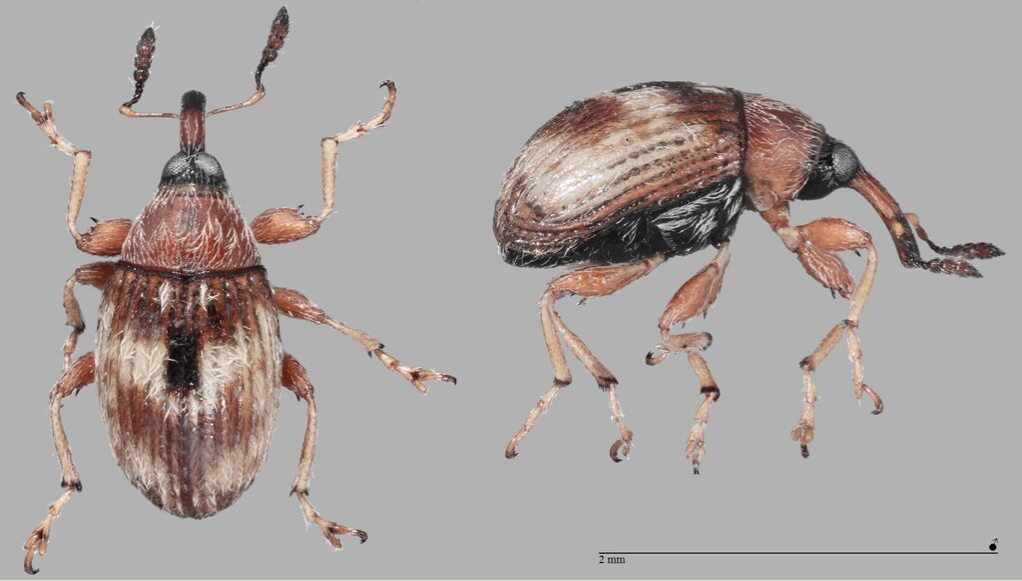
*Dieckmanniellusnitidulus* (Brentidae: Nanophyinae) (Credit: Peter E. Stüben).

*Dieckmanniellusnitidulus* (Brentidae: Nanophyinae) is mainly widespread in the Mediterranean Region and lives monophagously on various Lythraceae (e.g. *Lythrumsalicaria* L.). The record on Santa Maria (Azores) probably refers to *Lythrumborysthenicum* (Schrank) Litv. or *Lythrumjunceum* Banks & Solander. The species was also introduced on five of the seven Canary Islands, where it lives on *Lythrumhyssopifolia* L., on which one of us (PS) was able to reliably detect it on La Gomera. Characteristic features: Head, funicle and club of antennae mainly black; elytra in front of the white, V-shaped transverse mark with a dark spot on the front third of the sutural strip (see Fig. 7); 1.4 – 2.1 mm (Stüben 2022).

Gronopscf.fasciatus Küster, 1851

**Figure 8.**
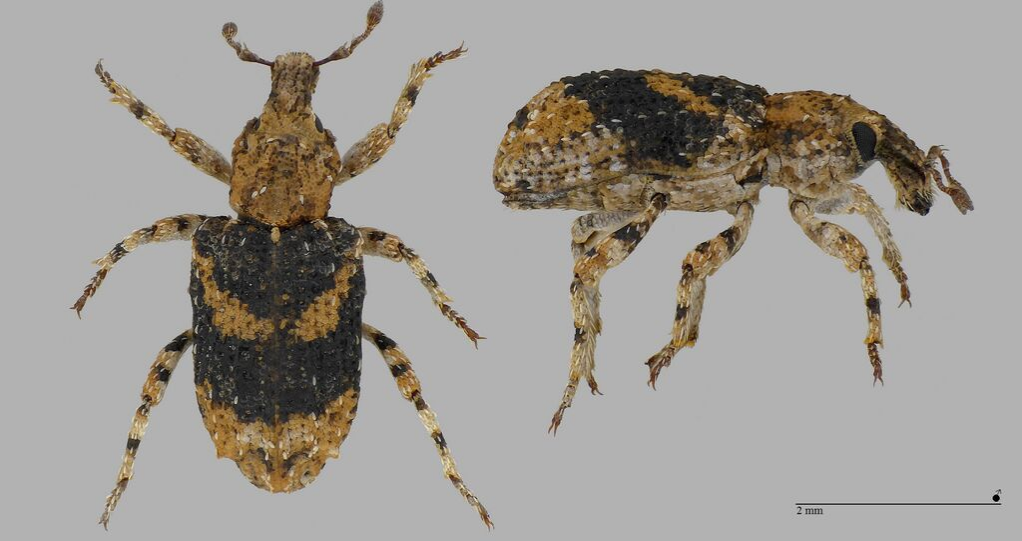
Gronopscf.fasciatus Küster, 1851 (Curculionidae: Cyclominae: Rhytirrhinini) (Credit: Peter E. Stüben).

The genus *Gronops* (Curculionidae: Cyclominae: Rhytirrhinini) includes about 20 Palaearctic species, mainly from the arid regions of North Africa and is only represented with certainty on the Canary Islands by the species *Gronopsfasciatus*. The determination of the specimen of *Gronopscf.fasciatus*, a male, recorded at the airfield of Santa Maria (Azores) in December 2019, must be checked again by a specialist of this group. The biology of these terricolous, flightless species is largely unknown, although they are often found near Caryophyllaceae and Amaranthaceae. Carry-over with soil is conceivable. Characteristic features of *G.fasciatus* compared to *G.lunatus* (Fabricius, 1775): elytra shorter, hardly narrowing towards the apex (subparallel); pronotum wider, strongly widened in the front third (see Fig. 8); length: 2.2–3.2 mm (Stüben 2022).

*Hadroplontustrimaculatus* (Fabricius, 1775)

**Figure 9.**
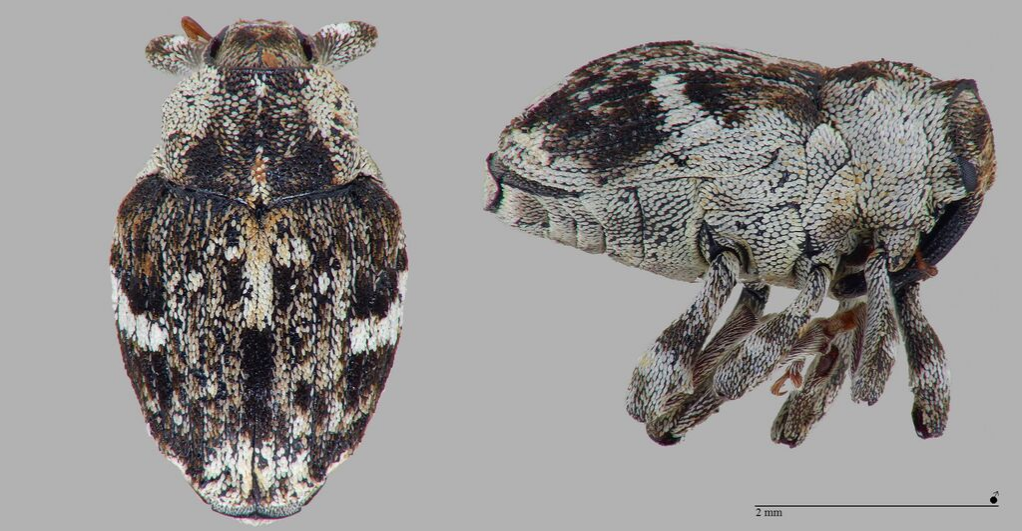
*Hadroplontustrimaculatus* (Fabricius, 1775) (Curculionidae: Ceutorhynchinae) (Credit: Peter E. Stüben).

Both European species, *Hadroplontuslitura* and *H.trimaculatus* (Curculionidae: Ceutorhynchinae) live on thistles. The latter lives on plants of the genus *Carduus*, mainly *C.nutans* and *C.acanthoides*. The single specimen (see Fig. 9) was sampled at Piquinhos, mixed forest with the presence of *Carduustenuiflorus* W.M.Curtis. *H.trimaculatus* differs from the very similar species *H.litura* by the beige to greyish-brown (not completely white) suture interval of the cruciform elytral spot (Stüben et al. 2014, Stüben 2022).

*Hypurusbertrandi* (Perris, 1852)

**Figure 10.**
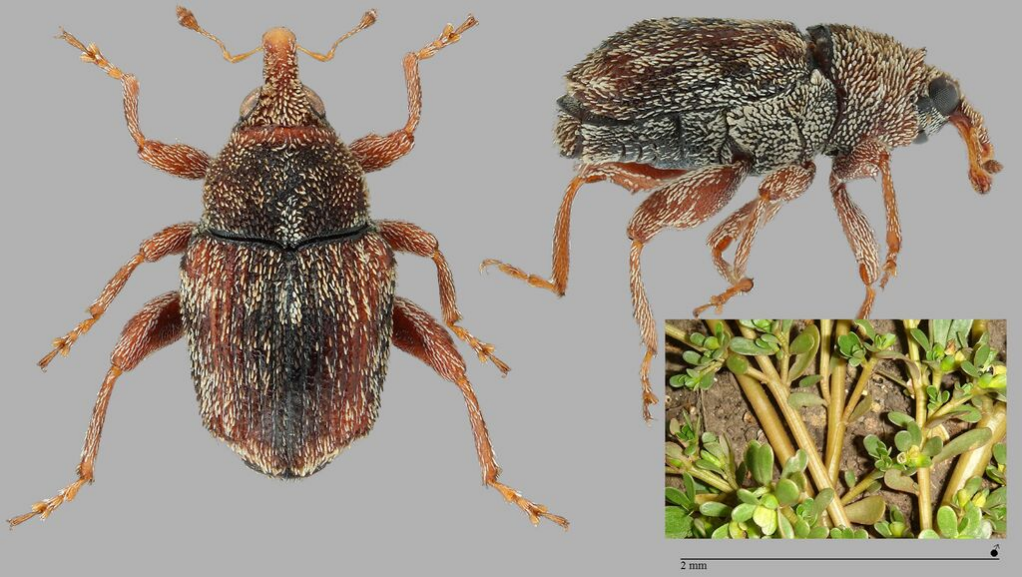
*Hypurusbertrandi* (Perris, 1852) (Curculionidae: Ceutorhynchinae) (Credit: Peter E. Stüben). The scale refers to the insect. The photo of the plants refers to *Portulacaoleracea* L., in which the species lives as monophagous.

*Hypurusbertrandi* (Perris, 1852) (Curculionidae: Ceutorhynchinae) (see Fig. 10), originally a Mediterranean, now nearly cosmopolitan species, which lives monophagously on *Portulacaoleracea* L. The species was first reported from the Macaronesian Islands by García et al. (2016), collected in the Escuela de Capacitación Agraria near Tacoronte on Tenerife in 2015. The species also occurs in Cape Verde (São Tiago: S. Jorge; Colonnelli 1990) and on the Azores (Terceira: Caldeira Lajes). A characteristic feature is the strongly thickened hind femora (see Fig. 10; Stüben et al. 2012, Stüben 2022).

*Cardiocondylamauritanica* Forel, 1890

**Figure 11.**
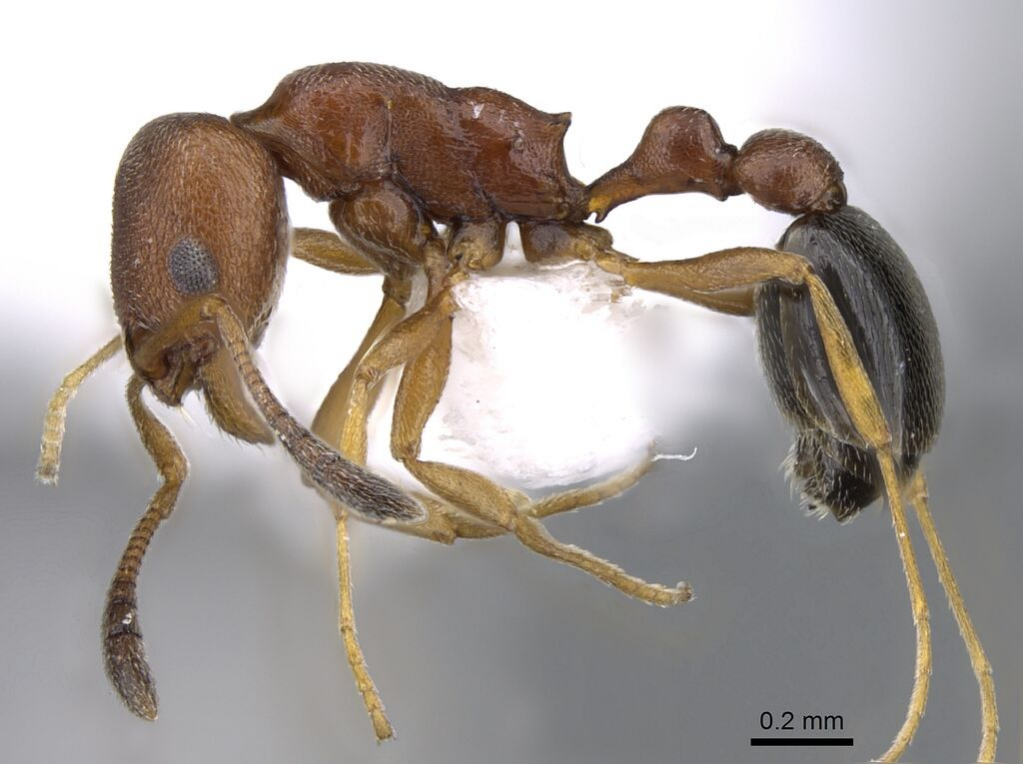
*Cardiocondylamauritanica* (Forel, 1890) (Formicidae). Specimen CASENT0746634 from AntWeb.org (Credit: Zach Lieberman).

This ant species is native to northern Africa, Middle East, Afghanistan and Pakistan, but has been introduced in many other regions, including the United States of America, Mexico, Zimbabwe, several European countries and many islands worldwide (Wetterer 2012, Janicki et al. 2016, Seifert et al. 2017). In Macaronesia, *C.mauritanica* was perviously known to occur in Madeira and the Canary Islands (Espadaler 2008, Báez and Oromí 2010). These ants are small, inconspicuous and can be separated from other *Cardiocondyla* species using a combination of morphometric characters (Seifert 2003, Seifert et al. 2017) (see Fig. 11). They form polygynous colonies and mating occurs inside the nests (Seifert et al. 2017). These characteristics and their ability to co-exist with other aggressive invasive ant species, like the Argentine ant *Linepithemahumile*, are important to explain their ecological success and ongoing spread (Wetterer 2012). However, contrary to other exotic ant species, *C.mauritanica* does not seem to have significant ecological impacts on native biodiversity (Wetterer 2012).

### Conservation remarks

This publication highlights the importance of exotic and mixed forest areas, as well as small native disturbed forest patches as potential reservoirs of both exotic potentially invasive species, as well as rare endemic species (see also Tsafack et al. 2021).

The high abundance of several native non-endemic (e.g. *Cyphopterumadcendens*; *Lasiusgrandis*, *Monomoriumcarbonarium*, *Leiobunumblackwalli*, *Trichopsocusclarus*) and endemic species (e.g. *Cixiusazoterceirae*, *Elipsocusbrincki*, *Elipsocusazoricus*, *Strophingiaharteni*) (Table 2) in these habitats is noteworthy.

**Figure 12.**
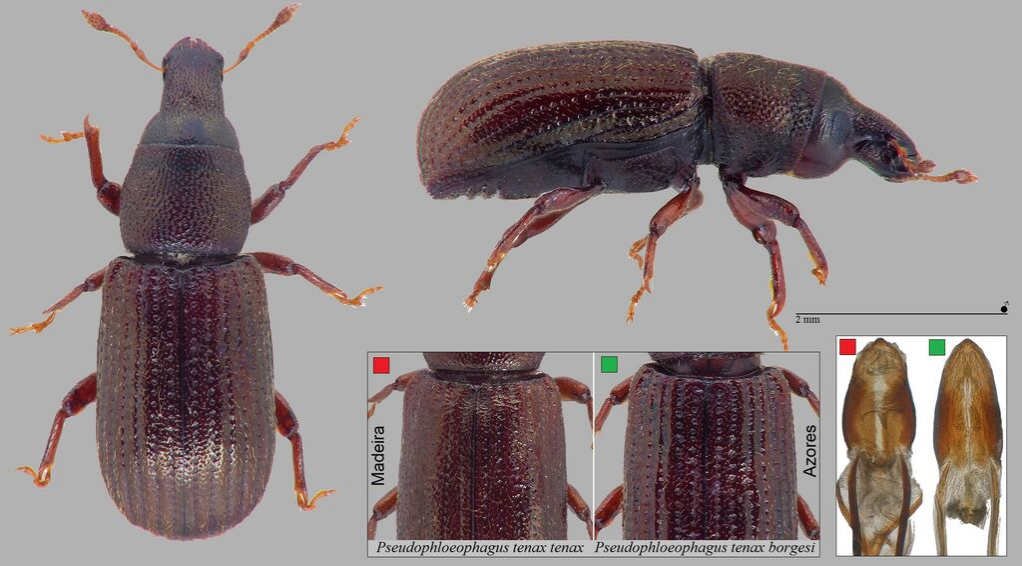
*Pseudophloeophagustenaxborgesi* Stüben, 2022 (Curculionidae: Cossoninae) (Credit: Peter E. Stüben).

Within endemics, we wish to comment on the recently-described subspecies *Pseudophloeophagustenaxborgesi* Stüben, 2022 (Curculionidae: Cossoninae) (Fig. 12). This subspecies, common in many islands of the Azores, was described only in 2022 (Stüben 2022). The nominotypic taxon occurs on Madeira. Accordingly, material of *P.tenax* from trap findings and in collections from the Azores must be assigned to this new subspecies. Type locality of *P.tenaxborgesi* is on São Jorge (Vigia da Baleia), but perhaps the subspecies occurs on all islands of the Azores. Apart from clear molecular differences in the mitochondrial COI gene (Stüben et al. 2012), this subspecies of the Azores differs from the nominotypic taxon on Madeira in the following characteristics: elytral striae more strongly and deeply punctured and the interstriae much narrower than in the sister taxon from Madeira; aedeagus narrower (see Fig. 12, Stüben and Borges 2019, Stüben 2022).

Several other rare endemic species were found in this study (see list below), which highlights the importance of expanding surveys in Azores to small isolated forest patches in order to find relict populations of rare endemic species:

- *Canariphantesacoreensis* (Wunderlich, 1992) (Araneae, Linyphiidae). This rare spider is usually found in pristine native forests and is considered Vulnerable (VU) by IUCN (Borges and Cardoso 2021b). In the current study, we sampled a single specimen in a disturbed mixed forest of *Eucalyptus* spp. and *Pittospsorumundulatum* at Terceira Island located near a native forest at Pico Rachado.

- *Canariphantesrelictus* Crespo & Bosmans, 2014 (Araneae, Linyphiidae). Another very rare species, classified as Critically Endangered (CR) (Borges and Cardoso 2021a). The species was found originally in high elevation at Santa Maria Island (Crespo et al. 2014), but in our study, two females were found at Piedade (PRIBS_T03_12_2019) at low elevation in a mixed forest of *Picconiaazorica*, *Pittosporumundulatum* and *Pinus* sp. The species possibly has a larger distribution than originally recorded.

-*Olisthopusinclavatus* Israelson, 1983 (Coleoptera, Carabidae). This is a very rare ground-beetle classified as Critically Endangered (CR) by IUCN (Borges 2018) and currently occurring only in exotic forests (dominated by *Cryptomeriajaponica*, *Acacia* spp.). In this study, the unique specimen was sampled in Monteiro (SMR_PRIBS_T12).

**Figure 13.**
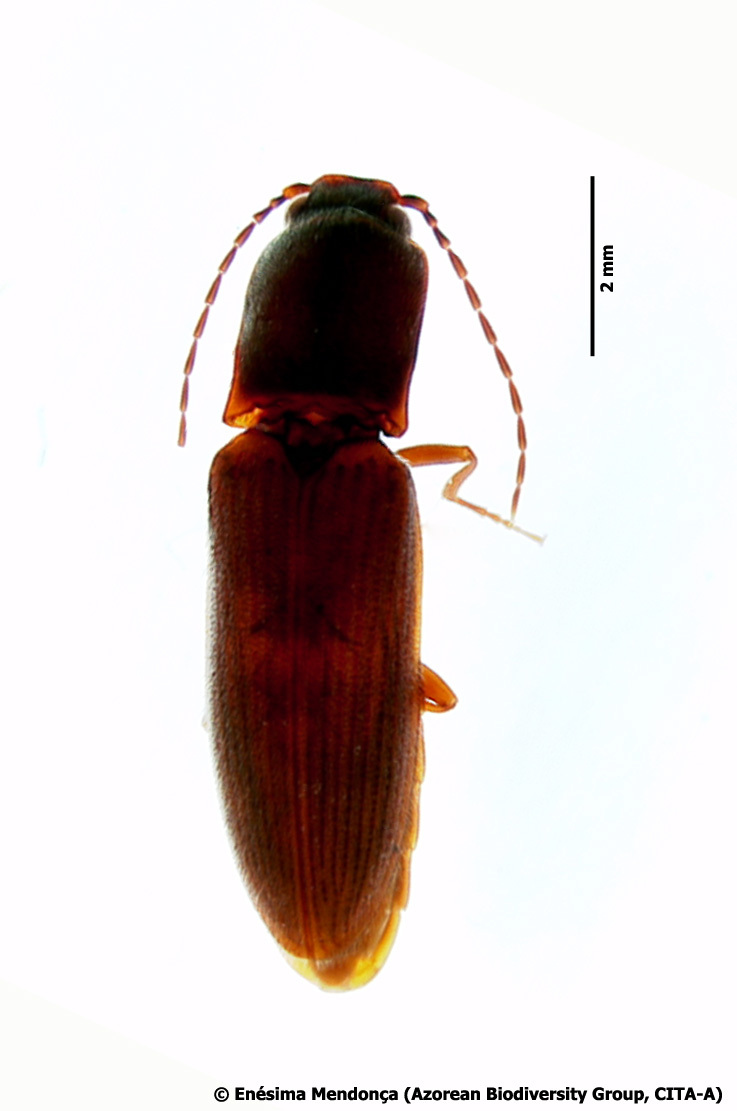
*Athousazoricus* Platia & Gudenzi, 2002 (Coleoptera, Elateridae) (Credit: Enésima Pereira, Azorean Biodiversity Group).

**Figure 14.**
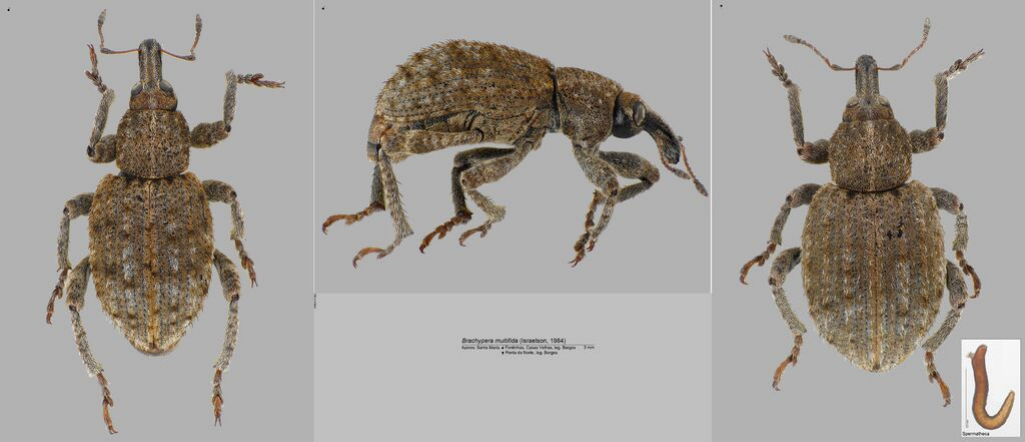
*Brachyperamultifida* (Israelson, 1984) (= *Donusmultifudus* (Israelson, 1984)) (Coleoptera, Curculionidae) (Credit: Peter E. Stüben).

**Figure 15.**
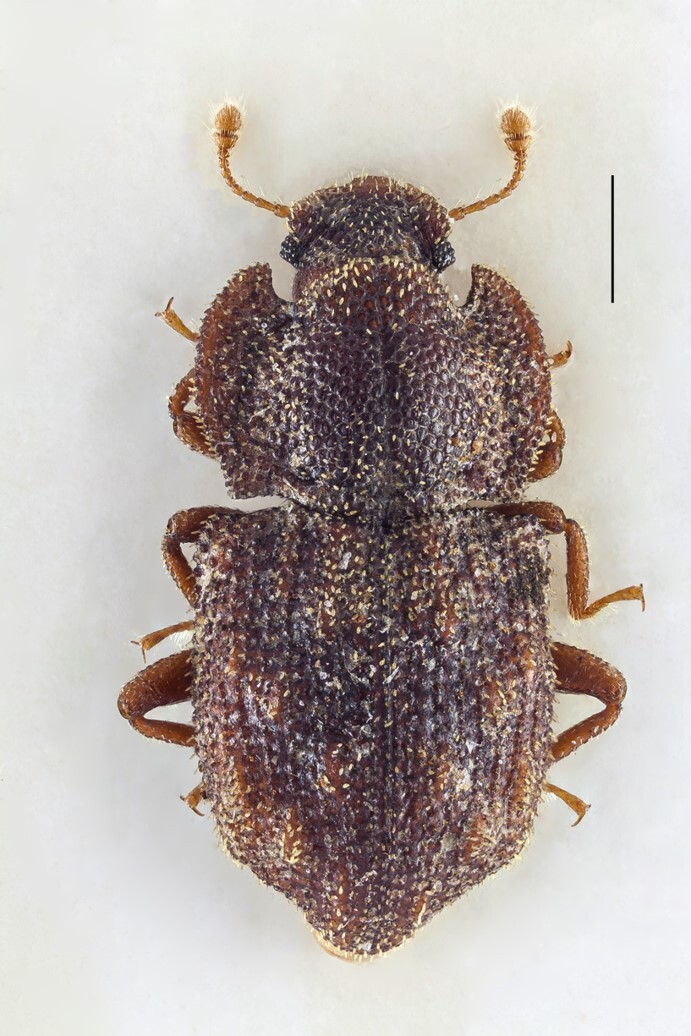
*Tarphiusrufonodulosus* Israelson, 1984 (Coleoptera, Zopheridae) (Credit: Erno-Endre Gergely; Azorean Biodiverity Group).

- *Athousazoricus* Platia & Gudenzi, 2002 (Coleoptera, Elateridae) (Fig. 13). This is a relatively rare species known from Flores, Graciosa, Terceira and S. Miguel Islands. Considered Endangered by the IUCN (Borges and Lamelas-López 2017a), this species tends to occur at low elevations in disturbed exotic forests. In the current study, we found the species in three places all at mid-elevations (300-500 m) and in three types of forest, one plantation of *Cryptomeriajaponica* (Mistérios Negros; TER-MNEG-T-62 corresponding also to code TER_PRIBS_T05), a mixed forest dominated by *Eucalyptus* spp. (Escampadouro; TER_PRIBS_T23) and a mixed forest dominated by *Pittosporumundulatum* (Mata do Estado; TER_PRIBS_T04). At least in Terceira Island, the species seems to be more widespread than previously assumed.

- *Brachyperamultifida* (Israelson, 1984) (= *Donusmultifudus* (Israelson, 1984)) (Coleoptera, Curculionidae) (Fig. 14). This is a particularly rare curculionid beetle classified as Critically Endangered (CR) (Borges and Lamelas-López 2017b). Previously, it was sampled at high elevation at Pico Alto in Santa Maria Island. In the current survey, we sampled this species in three sites, Estação Loran (SMR_PRIBS_T08), Piquinhos (SMR_PRIBS_T10) and Figueiral (SMR_PRIBS_T14) expanding the range of the species to lower elevations and to different types of forest.

- *Tarphiusrufonodulosus* Israelson, 1984 (Coleoptera, Zopheridae) (Fig. 15). This is a rare ironclade beetle also Critically Endangered (CR) (Borges and Lamelas-López 2018) that is associated with the canopies of native trees (e.g. *Picconiaazorica*) and under-bark of dead trees, both in native and exotic forests (dominated by *Acacia* sp. and *Cryptomeriajaponica*). In the current study, one specimen was collected in mixed forests of *Ericaazorica*, *Cryptomeriajaponica* and *Pittosporumundulatum* at three locations at high elevation (Piquinhos and Fontinhas forest areas).

### Patterns of invasion

The main aim of this study was to investigate the importance of disturbed native forest patches and exotic vegetation areas as potential reservoirs of exotic potentially invasive arthropods. As expected, we found a large number of exotic species, some of them new for Azores, as listed above. In addition to the 125 species identified as introduced (Table 2), many more are waiting a proper identification (Suppl. material 1). In previous studies, we identified thirteen widespread exotic arthropods as new records for Azores (Borges et al. 2013) and some previously unknown exotic species in Azorean urban gardens (Arteaga et al. 2020). This clearly indicates that there is an ongoing continuous flux of new introductions in Azores.

Some of the introduced species found in the current study are a matter of concern for nature conservation in the Azores Archipelago and their populations should be monitored. For instance, *Lagriahirta* (Linnaeus, 1758) (Coleoptera, Tenebrionidae) that was recently recorded as new for Azores and found originally at Terceira Island (Borges et al. 2021), is expanding dramatically in Santa Maria. In Santa Maria, we found it everywhere at all elevations and habitats. This seems to be a recent introduction in Azores and the impact of this species is still unknown. The Australian exotic planthopper *Siphantaacuta* (Walker, 1851) (Hemiptera, Flatidae) was recorded originally for Azores in 2013 (Borges et al. 2013) and is expanding rapidly in several Azorean islands with potential impacts on agriculture. In our study, we found it quite abundant in many sites at Terceira and Santa Maria Islands. The expansion of the Eucalyptus snout beetle *Gonipterusplatensis* (Marelli, 1926) (Coleoptera, Curculionidae) that was found in several sites at Terceira Island is also of concern. This species was originally recorded for Azores by Borges et al. (2013) and is currently known also from São Miguel Island.

In potential expansion in Terceira (and also known from Pico) is the two-spotted leafhopper *Sophoniaorientalis* (Matsumura, 1912) (Hemiptera; Cicadellidae) (Tarantino et al. 2022). This species is native to south-east Asia and is a highly polyphagous pest, considered an invasive species that affects crops as well as endemic plants (Tarantino et al. 2022).

Several exotic ant species have been recorded in the Azores (Espadaler 2010) and, here, we report new findings at both island and archipelago levels that highlight their rapid spread. Jointly with the first record of *Cardiocondylamauritanica* for Azores, we found that two exotic *Tetramorium* species (*T.bicarinatum* and *T.caldarium*) are now present in Santa Maria. Both *C.mauritanica* and *T.caldarium* do not seem to have significant impacts on native biodiversity (Wetterer 2012, Wetterer and Hita Garcia 2015), but *T.bicarinatum* and, particularly, the Argentine ant *L.humile*, are serious threats to island native invertebrates and natural ecological processes and have also been reported as agricultural pests (Wetterer 2009, Wetterer et al. 2009). The severe consequences of Argentine ant invasion on local biodiversity have been reported from many areas around the world, including oceanic islands, but their effects remain poorly understood in Macaronesian archipelagos (Holway et al. 2002, Wetterer and Espadaler 2010, Boieiro et al. 2018a, Boieiro et al. 2018b).

Finally, it is important to highlight that, amongst the most abundant introduced species in our study, several are listed in the TOP100 worst invasive species of Azores and Macaronesia (Silva et al. 2008), namely the woodlouse spider *Dysderacrocata* C.L. Koch, 1838, the Argentine ant *Linepithemahumile* (Mayr, 1868) and the millipede *Ommatoiulusmoreleti* (Lucas, 1860).

Our study stresses the need for arthropod biodiversity monitoring in different habitats of oceanic islands as an important strategy for early detection of invasive species that may have severe impacts on the environment, economy and human well-being (see also Borges et al. 2018). It also allows us to assess changes on species abundance and distribution, thus providing valuable information to support decision-making by conservation managers.

## Supplementary Material

C103BC9B-E17C-52DA-83C9-D6740B57F234 10.3897/BDJ.10.e81410.suppl1 Supplementary material 1 Complete list of species and morphospecies. Data type Excel Brief description Complete list of arthropod species collected in four islands of Azores, between 2019 and 2020 using SLAM traps. The list includes individuals identified at species-level and also Morphospecies. Abundance per island (COR - Corvo; FLO - Flores; TER - Terceira; SMR - Santa Maria) is provided. File: oo_639564.txt https://binary.pensoft.net/file/639564 Borges, P.A.V.

## Figures and Tables

**Table 1.** List of the 45 sampled sites in the Corvo (n = 1), Flores (n = 5), Santa Maria (n =16) and Terceira (n = 23) Islands. Information about LocationID, Locality, decimal coordinates and elevation in metres are provided.

Island	Habitat	Location ID	Locality	Latitude	Longitude	Elevation
Corvo	Mixed Forest - *Picconia*, *Pittosporum*	COR-CORO-Z-16	Coroa do Pico	39.68854	-31.09191	248
Flores	Exotic Forest - *Cryptomeria*	FLO-LAFLOR-T29	Lajes- Estação Florestal	39.39416	-31.20682	315
Flores	Exotic Forest - *Cryptomeria*	FLO-MAPS-TT25	Criptomérias ao lado do T16	39.48697	-31.18462	607
Flores	Native Forest	FLO-NFFR-T-07	Encosta Caldeira Funda	39.40324	-31.2175	381
Flores	Native Forest	FLO-NFMA-T-08	Morro Alto Este	39.46003	-31.20941	769
Flores	Mixed Forest	FLO-PDEL-Z-11	Ponta Delgada Km18_Mata das Acácias	39.50744	-31.2017	106
Santa Maria	Native Forest - *Erica*, *Picconia*	SMR_PRIBS_T01	Ponta do Pinheiro	37.00336	-25.12854	192
Santa Maria	Mixed Forest - *Erica*, *Picconia*, *Hedychium*	SMR_PRIBS_T02	Miradouro Pedra Rija	36.97597	-25.07578	355
Santa Maria	Mixed Forest - *Picconia*, *Pittosporum*, *Pinus*	SMR_PRIBS_T03	Piedade	36.93424	-25.0668	184
Santa Maria	Mixed Forest - *Laurus*, *Pittosporum*, *Picconia*, *Ilex*	SMR_PRIBS_T04	Setada	36.95356	-25.07398	374
Santa Maria	Native Forest - *Laurus*, *Erica*, *Ilex*	SMR_PRIBS_T05	Casas Velhas	36.95375	-25.07494	377
Santa Maria	Mixed Forest - *Picconia*, *Pittosporum*, *Erica*, *Hedychium*, *Vaccinium*	SMR_PRIBS_T06	Fontinhas Florestal	36.96325	-25.07505	406
Santa Maria	Mixed Forest - *Picconia*, *Pittosporum*	SMR_PRIBS_T07	Miradouro Espigão	36.98215	-25.0488	191
Santa Maria	Mixed Forest - *Erica*, *Picconia*, *Pittosporum*	SMR_PRIBS_T08	Estação Loran	37.00931	-25.05792	164
Santa Maria	Mixed Forest - *Erica*, *Cryptomeria*, *Hedychium*	SMR_PRIBS_T10	Piquinhos	36.97206	-25.08278	420
Santa Maria	Mixed Forest - *Picconia*, *Pittosporum*	SMR_PRIBS_T11	Lapa	36.94849	-25.02598	221
Santa Maria	Mixed Forest - *Acacia*, *Picconia*	SMR_PRIBS_T12	Monteiro	36.97013	-25.10942	191
Santa Maria	Exotic Forest - *Acacia*	SMR_PRIBS_T13	Aeroporto	36.97048	-25.1549	112
Santa Maria	Exotic Forest - *Pittosporum*	SMR_PRIBS_T14	Figueiral	36.94919	-25.12562	142
Santa Maria	Mixed Forest	SMR_PRIBS_T15	Ribeira dos Lemos	37.00141	-25.14769	61
Santa Maria	Native Forest - *Erica*, *Picconia*	SMR_PRIBS_T16	Caldeira	36.99592	-25.09864	304
Santa Maria	Mixed Forest - *Picconia*, *Erica*, *Laurus*, *Vaccinium*, *Hedychium*, *Myrcide*	SMR-NFPA-T-01 (SMR_PRIBS_T09)	Pico Alto T01	36.97804	-25.08756	460
Terceira	Exotic Forest - *Pittosporum*	TER_PRIBS_T02	Universidade	38.65868	-27.23262	43
Terceira	Exotic Forest - *Pittosporum*	TER_PRIBS_T04	Mata Estado Veredas	38.69814	-27.2421	450
Terceira	Exotic Forest - *Pittosporum*	TER_PRIBS_T07	Pittosporum Carpintaria dos Biscoitos	38.79017	-27.24136	93
Terceira	Exotic Forest - *Pittosporum*	TER_PRIBS_T08	Caldeira Lajes	38.77705	-27.11853	18
Terceira	Native Forest - *Juniperus*	TER_PRIBS_T11	Juniperal Trilho das Bestas	38.7087	-27.19133	521
Terceira	Native Forest - *Erica*	TER_PRIBS_T12	Erical Trilho das Bestas	38.70957	-27.18324	462
Terceira	Native Forest - *Juniperus*	TER_PRIBS_T13	Terra-Brava Rocha Cedrorum	38.74598	-27.19762	652
Terceira	Exotic Forest - *Cryptomeria*, *Calluna*	TER_PRIBS_T14	Pico Alto Cryptomeria_Calluna	38.75212	-27.20132	584
Terceira	Exotic Forest - *Pittosporum*, *Eucalyptus*	TER_PRIBS_T15	Eucaliptal Agualva	38.77109	-27.1934	344
Terceira	Exotic Forest - *Pittosporum*	TER_PRIBS_T17	Pittosporum_Eucalito Pizza-UT	38.75087	-27.07099	87
Terceira	Exotic Forest - *Pittosporum*	TER_PRIBS_T19	Pittosporum Maria Vieira	38.65377	-27.08076	102
Terceira	Exotic Forest - *Eucalyptus*	TER_PRIBS_T20	Ermida Penha França	38.66603	-27.23969	118
Terceira	Exotic Forest - *Eucalyptus*, *Acacia*	TER_PRIBS_T22	Eucalipto_Acacia_Canada Entre Picos	38.6615	-27.26605	78
Terceira	Exotic Forest - *Eucalyptus*, *Hedychium*	TER_PRIBS_T23	Eucalipto_Echinodium Escampador	38.70309	-27.27717	340
Terceira	Exotic Forest - *Pittosporum*, *Betula*	TER_PRIBS_T24	Betulas_Lagoa das Patas	38.71833	-27.28923	524
Terceira	Exotic Forest - *Eucalyptus*	TER_PRIBS_T27	Eucaliptal_Gruta Chocolate	38.77696	-27.25107	298
Terceira	Exotic Forest - *Pittosporum*, *Eucalyptus*	TER_PRIBS_T28	Eucaliptal_Pico Rachado_Altares	38.76211	-27.30704	522
Terceira	Mixed Forest - *Eucalyptus*, *Erica*	TER-ACAR-T-25 (TER_PRIBS_T10)	Eucaliptal Algar do Carvão	38.72638	-27.22258	530
Terceira	*Exotic* Forest - Pittosporum	TER-CABI-T166 (TER_PRIBS_T06)	Caparica - Biscoitos	38.77094	-27.26185	331
Terceira	Exotic Forest - *Acacia*	TER-FTER-T-36 (TER_PRIBS_T09)	Fontinhas 1	38.73765	-27.13681	245
Terceira	Native Forest - *Laurus*, *Erica*	TER-MATE-T-13 (TER_PRIBS_T01)	Matela 1	38.70063	-27.26074	392
Terceira	Exotic Forest - *Cryptomeria*	TER-MNEG-T-62 (TER_PRIBS_T05)	Lagoa do Negro	38.73977	-27.26341	571
Terceira	Exotic Forest - *Pittosporum*	TER-POSA-T172 (TER_PRIBS_T03)	Posto Santo	38.68365	-27.24457	246

**Table 2.** List of arthropod species collected in four islands of Azores, between 2019 and 2020 using SLAM traps. The list includes individuals identified at species-level. Scientific name, colonization status (CS: intr – introduced; nat - native non-endemic; end - endemic) and abundance per island (COR - Corvo; FLO - Flores; TER - Terceira; SMR - Santa Maria). Bold scientific names constitute new records for the Azores and bold numbers new records for a given island.

Class	Order	Family	Scientific Name	CS	COR	FLO	TER	SMR
Arachnida	Araneae	Agelenidae	*Tegenariapagana* C.L. Koch, 1840	intr			118	1
Arachnida	Araneae	Agelenidae	*Textrixcaudata* L. Koch, 1872	intr			3	
Arachnida	Araneae	Araneidae	*Agalenatearedii* (Scopoli, 1763)	intr		1		
Arachnida	Araneae	Araneidae	*Araneusangulatus* Clerck, 1757	intr			1	
Arachnida	Araneae	Araneidae	*Gibbaraneaoccidentalis* Wunderlich, 1989	end		5	85	3
Arachnida	Araneae	Araneidae	*Mangoraacalypha* (Walckenaer, 1802)	intr			1	
Arachnida	Araneae	Araneidae	*Zygiellax-notata* (Clerck, 1757)	intr			4	
Arachnida	Araneae	Cheiracanthiidae	*Cheiracanthiumerraticum* (Walckenaer, 1802)	intr			2	2
Arachnida	Araneae	Cheiracanthiidae	*Cheiracanthiumfloresense* Wunderlich, 2008	end		5		
Arachnida	Araneae	Cheiracanthiidae	*Cheiracanthiummildei* L. Koch, 1864	intr			**28**	39
Arachnida	Araneae	Clubionidae	*Clubionaterrestris* Westring, 1851	intr			6	1
Arachnida	Araneae	Clubionidae	*Porrhoclubionadecora* (Blackwall, 1859)	nat			158	43
Arachnida	Araneae	Clubionidae	*Porrhoclubionagenevensis* (L. Koch, 1866)	intr	**15**	19	41	47
Arachnida	Araneae	Dictynidae	*Emblynaacoreensis* Wunderlich, 1992	end			3	
Arachnida	Araneae	Dictynidae	*Lathysdentichelis* (Simon, 1883)	nat		2	16	7
Arachnida	Araneae	Dictynidae	*Nigmapuella* (Simon, 1870)	intr			6	1
Arachnida	Araneae	Dysderidae	*Dysderacrocata* C.L. Koch, 1838	intr		5	70	49
Arachnida	Araneae	Gnaphosidae	*Marinarozeloteslyonneti* (Audouin, 1826)	intr			1	
Arachnida	Araneae	Linyphiidae	*Acorigoneacoreensis* (Wunderlich, 1992)	end		1	35	1
Arachnida	Araneae	Linyphiidae	*Agynetafuscipalpa* (C. L. Koch, 1836)	intr			11	
Arachnida	Araneae	Linyphiidae	*Canariphantesacoreensis* (Wunderlich, 1992)	end			1	
Arachnida	Araneae	Linyphiidae	*Canariphantesrelictus* Crespo & Bosmans, 2014	end				2
Arachnida	Araneae	Linyphiidae	*Erigoneautumnalis* Emerton, 1882	intr			12	3
Arachnida	Araneae	Linyphiidae	*Erigonedentipalpis* (Wider, 1834)	intr			2	1
Arachnida	Araneae	Linyphiidae	*Lessertiadentichelis* (Simon, 1884)	intr				**3**
Arachnida	Araneae	Linyphiidae	*Microlinyphiajohnsoni* (Blackwall, 1859)	nat			348	
Arachnida	Araneae	Linyphiidae	*Miniciafloresensis* Wunderlich, 1992	end			2	
Arachnida	Araneae	Linyphiidae	*Nerieneclathrata* (Sundevall, 1830)	intr	**2**		1	
Arachnida	Araneae	Linyphiidae	*Oedothoraxfuscus* (Blackwall, 1834)	intr			8	
Arachnida	Araneae	Linyphiidae	*Palliduphantesschmitzi* (Kulczynski, 1899)	nat			2	
Arachnida	Araneae	Linyphiidae	*Pelecopsisparallela* (Wider, 1834)	intr			2	
Arachnida	Araneae	Linyphiidae	*Prinerigonevagans* (Audouin, 1826)	intr			1	
Arachnida	Araneae	Linyphiidae	*Savigniorrhipisacoreensis* Wunderlich, 1992	end		30	197	7
Arachnida	Araneae	Linyphiidae	*Tenuiphantesmiguelensis* (Wunderlich, 1992)	nat		3	16	
Arachnida	Araneae	Linyphiidae	*Tenuiphantestenuis* (Blackwall, 1852)	intr	6	17	346	21
Arachnida	Araneae	Mimetidae	*Erofurcata* (Villers, 1789)	intr			10	
Arachnida	Araneae	Oecobiidae	*Oecobiusnavus* Blackwall, 1859	intr			13	
Arachnida	Araneae	Pisauridae	*Pisauraacoreensis* Wunderlich, 1992	end			57	
Arachnida	Araneae	Salticidae	*Macaroeriscata* (Blackwall, 1867)	nat			56	
Arachnida	Araneae	Salticidae	*Macaroerisdiligens* (Blackwall, 1867)	nat		6	17	3
Arachnida	Araneae	Salticidae	*Neonacoreensis* Wunderlich, 2008	end				1
Arachnida	Araneae	Salticidae	*Phidippusaudax* (Hentz, 1845)	intr				3
Arachnida	Araneae	Salticidae	*Pseudeuophrysvafra* (Blackwall, 1867)	intr				1
Arachnida	Araneae	Salticidae	*Salticusmutabilis* Lucas, 1846	intr			3	
Arachnida	Araneae	Segestriidae	*Segestriaflorentina* (Rossi, 1790)	intr			10	1
Arachnida	Araneae	Tetragnathidae	*Metellinamerianae* (Scopoli, 1763)	intr			12	
Arachnida	Araneae	Tetragnathidae	*Sancusacoreensis* (Wunderlich, 1992)	end		1	10	
Arachnida	Araneae	Theridiidae	*Cryptachaeablattea* (Urquhart, 1886)	intr			31	
Arachnida	Araneae	Theridiidae	*Lasaeolaoceanica* Simon, 1883	end			2	1
Arachnida	Araneae	Theridiidae	*Rugathodesacoreensis* Wunderlich, 1992	end		7	81	7
Arachnida	Araneae	Theridiidae	*Steatodagrossa* (C. L. Koch, 1838)	intr			1	1
Arachnida	Araneae	Theridiidae	*Steatodanobilis* (Thorell, 1875)	intr			10	10
Arachnida	Araneae	Theridiidae	*Theridionmusivivum* Schmidt, 1956	nat			9	3
Arachnida	Araneae	Thomisidae	*Xysticuscor* Canestrini, 1873	nat			1	
Arachnida	Opiliones	Leiobunidae	*Leiobunumblackwalli* Meade, 1861	nat		13	857	**3**
Arachnida	Opiliones	Sclerosomatidae	*Homalenotuscoriaceus* (Simon, 1879)	nat		1	24	
Arachnida	Pseudoscorpiones	Chthoniidae	*Chthoniusischnocheles* (Hermann, 1804)	intr			7	
Arachnida	Pseudoscorpiones	Chthoniidae	*Ephippiochthoniustetrachelatus* (Preyssler, 1790)	intr	11		37	29
Arachnida	Pseudoscorpiones	Neobisiidae	*Neobisiummaroccanum* Beier, 1930	intr		1	13	
Chilopoda	Geophilomorpha	Linotaeniidae	*Strigamiacrassipe*s (C.L. Koch, 1835)	nat			3	
Chilopoda	Lithobiomorpha	Lithobiidae	*Lithobiuspilicornispilicornis* Newport, 1844	nat		1	37	
Chilopoda	Scolopendromorpha	Cryptopidae	*Cryptopshortensis* (Donovan, 1810)	nat			1	
Chilopoda	Scutigeromorpha	Scutigeridae	*Scutigeracoleoptrata* (Linnaeus, 1758)	intr	**1**	**4**	27	148
Diplopoda	Julida	Blaniulidae	*Blaniulusguttulatus* (Fabricius, 1798)	intr			1	
Diplopoda	Julida	Blaniulidae	*Nopoiuluskochii* (Gervais, 1847)	intr			4	
Diplopoda	Julida	Julidae	*Ommatoiulusmoreleti* (Lucas, 1860)	intr		38	166	23
Diplopoda	Polydesmida	Paradoxosomatidae	*Oxidusgracilis* (C.L. Koch, 1847)	intr		1	1	1
Diplopoda	Polydesmida	Polydesmidae	*Polydesmuscoriaceus* Porat, 1870	intr			5	1
Insecta	Archaeognatha	Machilidae	*Diltasaxicola* (Womersley, 1930)	nat		8	582	16
Insecta	Archaeognatha	Machilidae	*Trigoniophthalmusborgesi* Mendes, Gaju, Bach & Molero, 2000	end			15	
Insecta	Blattodea	Blattellidae	*Lobopteradecipiens* (Germar, 1817)	nat				1
Insecta	Blattodea	Corydiidae	*Zethasimonyi* (Krauss, 1892)	nat		8	127	79
Insecta	Coleoptera	Anthicidae	*Hirticollisquadriguttatus* (Rossi, 1792)	nat			18	
Insecta	Coleoptera	Apionidae	*Aspidapionradiolus* (Marsham, 1802)	nat			6	25
Insecta	Coleoptera	Apionidae	*Kalcapionsemivittatumsemivittatum* (Gyllenhal, 1833)	nat			27	1
Insecta	Coleoptera	Brentidae	***Dieckmanniellusnitidulus* (Gyllenhal, 1838)**	intr				**5**
Insecta	Coleoptera	Carabidae	*Anisodactylusbinotatus* (Fabricius, 1787)	intr			1	
Insecta	Coleoptera	Carabidae	*Dromiusmeridionalis* Dejean, 1825	intr			11	2
Insecta	Coleoptera	Carabidae	*Notiophilusquadripunctatus* Dejean, 1826	nat				1
Insecta	Coleoptera	Carabidae	*Olisthopusinclavatus* Israelson, 1983	end				1
Insecta	Coleoptera	Carabidae	*Stenolophusteutonus* (Schrank, 1781)	nat			2	
Insecta	Coleoptera	Cerambycidae	*Monochamusgalloprovincialis* (Olivier, 1795)	intr				**1**
Insecta	Coleoptera	Chrysomelidae	*Chaetocnemahortensis* (Fourcroy, 1785)	intr			4	1
Insecta	Coleoptera	Chrysomelidae	*Chrysolinabankii* (Fabricius, 1775)	nat				1
Insecta	Coleoptera	Chrysomelidae	*Epitrixcucumeris* (Harris, 1851)	intr			7	1
Insecta	Coleoptera	Chrysomelidae	*Epitrixhirtipennis* (Melsheimer, 1847)	intr		**1**	4	1
Insecta	Coleoptera	Chrysomelidae	*Longitarsuskutscherae* (Rye, 1872)	intr		1	1	38
Insecta	Coleoptera	Chrysomelidae	*Psylliodeschrysocephalus* (Linnaeus, 1758)	intr		1		8
Insecta	Coleoptera	Chrysomelidae	*Psylliodesmarcida* (Illiger, 1807)	nat	**1**		3	40
Insecta	Coleoptera	Coccinellidae	*Clitostethusarcuatus* (Rossi, 1794)	intr			1	
Insecta	Coleoptera	Coccinellidae	*Noviuscardinalis* (Mulsant, 1850)	intr		9	3	2
Insecta	Coleoptera	Coccinellidae	*Rhyzobiuslitura* (Fabricius, 1787)	nat			1	
Insecta	Coleoptera	Coccinellidae	*Rhyzobiuslophanthae* (Blaisdell, 1892)	intr			1	1
Insecta	Coleoptera	Coccinellidae	*Scymnusinterruptus* (Goeze, 1777)	nat			5	2
Insecta	Coleoptera	Coccinellidae	*Stethoruspusillus* (Herbst, 1797)	nat			12	
Insecta	Coleoptera	Corylophidae	*Sericoderuslateralis* (Gyllenhal, 1827)	intr			89	172
Insecta	Coleoptera	Cryptophagidae	*Cryptophaguscellaris* (Scopoli, 1763)	intr			1	3
Insecta	Coleoptera	Curculionidae	*Brachyperamultifida* (Israelson, 1984)	end				7
Insecta	Coleoptera	Curculionidae	*Brachytemnusporcatus* (Germar, 1823)	intr			2	
Insecta	Coleoptera	Curculionidae	*Calacallessubcarinatus* (Israelson, 1984)	end		19	86	19
Insecta	Coleoptera	Curculionidae	*Cathormioceruscurvipes* (Wollaston, 1854)	nat				4
Insecta	Coleoptera	Curculionidae	*Charagmusgressorius* (Fabricius, 1792)	intr			1	7
Insecta	Coleoptera	Curculionidae	*Coccotrypescarpophagus* (Hornung, 1842)	intr			7	
Insecta	Coleoptera	Curculionidae	*Dichromacallesdromedariu*s (Boheman, 1844)	intr				1
Insecta	Coleoptera	Curculionidae	*Gonipterusplatensis* (Marelli, 1926)	intr			47	
Insecta	Coleoptera	Curculionidae	***Gronopsfasciatus* Küster, 1851**	intr				**4**
Insecta	Coleoptera	Curculionidae	***Hadroplontustrimaculatus* (Fabricius, 1775)**	intr				**1**
Insecta	Coleoptera	Curculionidae	***Hypurusbertrandi* (Perris, 1852)**	intr			**1**	
Insecta	Coleoptera	Curculionidae	*Mecinuspascuorum* (Gyllenhal, 1813)	intr			17	4
Insecta	Coleoptera	Curculionidae	*Mogulonesgeographicus* (Goeze, 1777)	intr				2
Insecta	Coleoptera	Curculionidae	*Naupactuscervinus* (Boheman, 1840)	intr			49	23
Insecta	Coleoptera	Curculionidae	*Otiorhynchuscribricollis* Gyllenhal, 1834	intr			19	4
Insecta	Coleoptera	Curculionidae	*Otiorhynchusrugosostriatus* (Goeze, 1777)	intr			1	
Insecta	Coleoptera	Curculionidae	*Pseudophloeophagustenaxborgesi* Stüben, 2022	nat	1	4	260	12
Insecta	Coleoptera	Curculionidae	*Rhopalomesitestardyi* (Curtis, 1825)	nat		2	4	
Insecta	Coleoptera	Curculionidae	*Sitonadiscoideus* Gyllenhal, 1834	intr			9	
Insecta	Coleoptera	Curculionidae	*Sitonalineatus* (Linnaeus, 1758)	intr			1	
Insecta	Coleoptera	Curculionidae	*Xyleborinusalni* Nijima, 1909	intr			2	
Insecta	Coleoptera	Dryopidae	*Dryopsalgiricus* (Lucas, 1846)	nat			2	1
Insecta	Coleoptera	Dryopidae	*Dryopsluridus* (Erichson, 1847)	nat			18	
Insecta	Coleoptera	Elateridae	*Aeolusmelliculusmoreleti* Tarnier, 1860	intr				1
Insecta	Coleoptera	Elateridae	*Athousazoricus* Platia & Gudenzi, 2002	end			21	
Insecta	Coleoptera	Elateridae	*Heteroderesazoricus* (Tarnier, 1860)	end		11	30	47
Insecta	Coleoptera	Elateridae	*Heteroderesvagus* Candèze, 1893	intr			2	
Insecta	Coleoptera	Elateridae	*Melanotusdichrous* (Erichson, 1841)	intr		1		
Insecta	Coleoptera	Histeridae	*Carcinopspumilio* (Erichson, 1834)	intr			1	
Insecta	Coleoptera	Hydrophilidae	*Cercyonhaemorrhoidalis* (Fabricius, 1775)	intr			5	
Insecta	Coleoptera	Latridiidae	*Cartoderebifasciata* (Reitter, 1877)	intr			21	1
Insecta	Coleoptera	Latridiidae	*Cartoderenodifer* (Westwood, 1839)	intr			16	1
Insecta	Coleoptera	Latridiidae	*Metophthalmusoccidentalis* Israelson, 1984	end			15	
Insecta	Coleoptera	Leiodidae	*Catopscoracinus* Kellner, 1846	nat			80	33
Insecta	Coleoptera	Malachiidae	*Attaluslusitanicuslusitanicus* Erichson, 1840	nat			4	
Insecta	Coleoptera	Mycetophagidae	*Litargusbalteatus* Le Conte, 1856	intr			3	
Insecta	Coleoptera	Mycetophagidae	*Typhaeastercorea* (Linnaeus, 1758)	intr			3	1
Insecta	Coleoptera	Nitidulidae	*Brassicogethesaeneus* (Fabricius, 1775)	intr			6	
Insecta	Coleoptera	Nitidulidae	*Carpophilusfumatus* Boheman, 1851	intr			1	
Insecta	Coleoptera	Nitidulidae	*Epuraeabiguttata* (Thunberg, 1784)	intr		1		3
Insecta	Coleoptera	Nitidulidae	*Phenolialimbatatibialis* (Boheman, 1851)	intr			2	
Insecta	Coleoptera	Nitidulidae	*Stelidotageminata* (Say, 1825)	intr			19	106
Insecta	Coleoptera	Phalacridae	*Stilbustestaceus* (Panzer, 1797)	nat			16	25
Insecta	Coleoptera	Ptiliidae	*Ptenidiumpusillum* (Gyllenhal, 1808)	intr			3	47
Insecta	Coleoptera	Ptinidae	*Anobiumpunctatum* (De Geer, 1774)	intr	2		69	
Insecta	Coleoptera	Ptinidae	*Calymmaderussolidus* (Kiesenwetter, 1877)	intr			1	
Insecta	Coleoptera	Rutelidae	*Popilliajaponica* Newman, 1838	intr		**1**	6	
Insecta	Coleoptera	Scraptiidae	*Anaspisproteus* Wollaston, 1854	nat	1	24	290	1
Insecta	Coleoptera	Silvanidae	*Cryptamorphadesjardinsii* (Guérin-Méneville, 1844)	intr				1
Insecta	Coleoptera	Staphylinidae	*Aleocharabipustulata* (Linnaeus, 1760)	intr			19	12
Insecta	Coleoptera	Staphylinidae	*Aloconotasulcifrons* (Stephens, 1832)	nat			38	1
Insecta	Coleoptera	Staphylinidae	*Amischaanalis* (Gravenhorst, 1802)	intr			1	
Insecta	Coleoptera	Staphylinidae	*Astenuslyonessius* (Joy, 1908)	nat			2	3
Insecta	Coleoptera	Staphylinidae	*Athetaaeneicollis* (Sharp, 1869)	intr			17	9
Insecta	Coleoptera	Staphylinidae	*Athetafungi* (Gravenhorst, 1806)	intr	**5**	1	358	282
Insecta	Coleoptera	Staphylinidae	*Carpelimuscorticinus* (Gravenhorst, 1806)	nat			2	2
Insecta	Coleoptera	Staphylinidae	*Coproporuspulchellus* (Erichson, 1839)	intr			5	
Insecta	Coleoptera	Staphylinidae	*Cordaliaobscura* (Gravenhorst, 1802)	intr			2	
Insecta	Coleoptera	Staphylinidae	*Euconnusazoricus* Franz, 1969	end			1	**2**
Insecta	Coleoptera	Staphylinidae	*Gyrohypnusfracticornis* (Müller, 1776)	intr			2	
Insecta	Coleoptera	Staphylinidae	*Hypomedondebilicornis* (Wollaston, 1857)	nat				1
Insecta	Coleoptera	Staphylinidae	*Notothectadryochares* (Israelson, 1985)	end			1	
Insecta	Coleoptera	Staphylinidae	*Ocypusaethiops* (Waltl, 1835)	nat			1	
Insecta	Coleoptera	Staphylinidae	*Oligotapumilio* Kiesenwetter, 1858	nat			5	4
Insecta	Coleoptera	Staphylinidae	*Phloeonomuspunctipennis* Thomson, 1867	nat			1	
Insecta	Coleoptera	Staphylinidae	*Proteinusatomarius* Erichson, 1840	nat			4	2
Insecta	Coleoptera	Staphylinidae	*Rugilusorbiculatus* (Paykull, 1789)	nat			2	1
Insecta	Coleoptera	Staphylinidae	*Sepedophiluslusitanicus* Hammond, 1973	nat			1	
Insecta	Coleoptera	Staphylinidae	*Stenomastaxmadeirae* Assing, 2003	nat				**4**
Insecta	Coleoptera	Staphylinidae	*Tachyporuschrysomelinus* (Linnaeus, 1758)	intr		1	22	14
Insecta	Coleoptera	Staphylinidae	*Tachyporusnitidulus* (Fabricius, 1781)	intr			64	42
Insecta	Coleoptera	Staphylinidae	*Trichiusaimmigrata* Lohse, 1984	intr			1	
Insecta	Coleoptera	Staphylinidae	*Trichophyapilicornis* (Gyllenhal, 1810)	nat				**1**
Insecta	Coleoptera	Staphylinidae	*Xantholinuslongiventris* Heer, 1839	intr			1	
Insecta	Coleoptera	Tenebrionidae	*Lagriahirta* (Linnaeus, 1758)	intr				**382**
Insecta	Coleoptera	Teredidae	*Anommatusduodecimstriatus* (Müller, 1821)	intr				2
Insecta	Coleoptera	Zopheridae	*Tarphiusrufonodulosus* Israelson, 1984	end				6
Insecta	Dermaptera	Anisolabididae	*Euborelliaannulipes* (Lucas, 1847)	intr				7
Insecta	Dermaptera	Forficulidae	*Forficulaauricularia* Linnaeus, 1758	intr			58	17
Insecta	Hemiptera	Anthocoridae	*Anthocorisnemoralis* (Fabricius, 1794)	nat			1	**3**
Insecta	Hemiptera	Anthocoridae	*Brachystelesparvicornis* (A. Costa, 1847)	nat			2	
Insecta	Hemiptera	Anthocoridae	*Buchananiellacontinua* (White, 1880)	intr			2	
Insecta	Hemiptera	Anthocoridae	*Oriuslaevigatuslaevigatus* (Fieber, 1860)	nat		2	4	15
Insecta	Hemiptera	Aphididae	*Cinarajuniperi* (De Geer, 1773)	nat	4	24	252	
Insecta	Hemiptera	Cicadellidae	*Aphrodeshamiltoni* Quartau & Borges, 2003	end			4	1
Insecta	Hemiptera	Cicadellidae	*Eupteryxazorica* Ribaut, 1941	end	14	64	107	23
Insecta	Hemiptera	Cicadellidae	*Eupteryxfilicum* (Newman, 1853)	nat			39	43
Insecta	Hemiptera	Cicadellidae	*Euscelidiusvariegatus* (Kirschbaum, 1858)	nat			2	3
Insecta	Hemiptera	Cicadellidae	*Sophoniaorientalis* (Matsumura, 1912)	intr			4	
Insecta	Hemiptera	Cixiidae	*Cixiusazofloresi* Remane & Asche, 1979	end		45		
Insecta	Hemiptera	Cixiidae	*Cixiusazomariae* Remane & Asche, 1979	end				278
Insecta	Hemiptera	Cixiidae	*Cixiusazoterceirae* Remane & Asche, 1979	end			968	
Insecta	Hemiptera	Delphacidae	*Kelisiaribauti* Wagner, 1938	nat			21	8
Insecta	Hemiptera	Flatidae	*Cyphopterumadcendens* (Herrich-Schäffer, 1835)	nat	1	44	1613	49
Insecta	Hemiptera	Flatidae	*Siphantaacuta* (Walker, 1851)	intr			136	**260**
Insecta	Hemiptera	Liviidae	*Strophingiaharteni* Hodkinson, 1981	end			340	192
Insecta	Hemiptera	Lygaeidae	*Kleidocerysericae* (Horváth, 1908)	nat			138	11
Insecta	Hemiptera	Lygaeidae	*Nysiusatlantidum* Horváth, 1890	end		1	2	
Insecta	Hemiptera	Microphysidae	*Loriculaelegantula* (Bärensprung, 1858)	nat		2	77	76
Insecta	Hemiptera	Miridae	*Campyloneuravirgul*a (Herrich-Schaeffer, 1835)	nat		1	128	
Insecta	Hemiptera	Miridae	*Heterotomaplanicornis* (Pallas, 1772)	nat			1	
Insecta	Hemiptera	Miridae	*Monalocorisfilicis* (Linnaeus, 1758)	nat		8	100	5
Insecta	Hemiptera	Miridae	*Pilophorusconfusus* (Kirschbaum, 1856)	nat			13	3
Insecta	Hemiptera	Miridae	*Pilophorusperplexus* Douglas & Scott, 1875	nat			1	
Insecta	Hemiptera	Miridae	*Pinalitusoromii* J. Ribes, 1992	end		1	18	35
Insecta	Hemiptera	Miridae	*Taylorilygusapicalis* (Fieber, 1861)	intr			1	
Insecta	Hemiptera	Nabidae	*Nabispseudoferusibericus* Remane, 1962	nat			11	7
Insecta	Hemiptera	Pentatomidae	*Piezodoruslituratus* (Fabricius, 1794)	nat			1	
Insecta	Hemiptera	Psyllidae	*Acizziauncatoides* (Ferris & Klyver, 1932)	intr			288	**23**
Insecta	Hemiptera	Reduviidae	*Empicorisrubromaculatus* (Blackburn, 1889)	intr			2	3
Insecta	Hemiptera	Rhyparochromidae	*Aphanusrolandri* (Linnaeus, 1758)	nat			1	
Insecta	Hemiptera	Rhyparochromidae	*Beosusmaritimus* (Scopoli, 1763)	nat				2
Insecta	Hemiptera	Rhyparochromidae	*Emblethisdenticollis* Horváth, 1878	nat			1	
Insecta	Hemiptera	Rhyparochromidae	*Eremocorismaderensis* (Wollaston, 1858)	nat			**1**	13
Insecta	Hemiptera	Rhyparochromidae	*Plinthisusbrevipennis* (Latreille, 1807)	nat				2
Insecta	Hemiptera	Rhyparochromidae	*Plinthisusminutissimus* Fieber, 1864	nat			1	
Insecta	Hemiptera	Rhyparochromidae	*Scolopostethusdecoratus* (Hahn, 1833)	nat		2	3	71
Insecta	Hemiptera	Saldidae	*Saldulapalustris* (Douglas, 1874)	nat			2	
Insecta	Hemiptera	Tingidae	*Tingisauriculata* (A. Costa, 1847)	intr				1
Insecta	Hemiptera	Triozidae	*Triozalaurisilvae* Hodkinson, 1990	nat			56	59
Insecta	Hymenoptera	Apidae	*Bombusruderatus* (Fabricius, 1775)	intr			6	
Insecta	Hymenoptera	Apidae	*Bombusterrestris* (Linnaeus, 1758)	intr		1	61	2
Insecta	Hymenoptera	Formicidae	***Cardiocondylamauritanica* Forel, 1890**	intr			**1**	**4**
Insecta	Hymenoptera	Formicidae	*Hypoponeraeduardi* (Forel, 1894)	nat	**1**	1	23	173
Insecta	Hymenoptera	Formicidae	*Hypoponerapunctatissima* (Roger, 1859)	intr				2
Insecta	Hymenoptera	Formicidae	*Lasiusgrandis* Forel, 1909	nat	31	38	856	348
Insecta	Hymenoptera	Formicidae	*Linepithemahumile* (Mayr, 1868)	intr			55	199
Insecta	Hymenoptera	Formicidae	*Monomoriumcarbonarium* (F. Smith, 1858)	nat			786	310
Insecta	Hymenoptera	Formicidae	*Plagiolepisschmitzii* Forel, 1895	nat			**18**	343
Insecta	Hymenoptera	Formicidae	*Temnothoraxunifasciatus* (Latreille, 1798)	nat			**18**	
Insecta	Hymenoptera	Formicidae	*Tetramoriumbicarinatum* (Nylander, 1846)	intr				**230**
Insecta	Hymenoptera	Formicidae	*Tetramoriumcaespitum* (Linnaeus, 1758)	nat			52	4
Insecta	Hymenoptera	Formicidae	*Tetramoriumcaldarium* (Roger, 1857)	intr			121	**2**
Insecta	Isoptera	Kalotermitidae	*Kalotermesflavicollis* (Fabricius, 1793)	intr			10	
Insecta	Neuroptera	Hemerobiidae	*Hemerobiusazoricus* Tjeder, 1948	end		2	31	15
Insecta	Orthoptera	Gryllidae	*Eumodicogryllusbordigalensis* (Latreille, 1804)	intr				1
Insecta	Orthoptera	Tettigoniidae	*Phaneropteranana* Fieber, 1853	nat			1	
Insecta	Phasmatodea	Phasmatidae	*Carausiusmorosus* (Sinéty, 1901)	intr			1	3
Insecta	Psocodea	Caeciliusidae	*Valenzuelaburmeisteri* (Brauer, 1876)	nat			136	35
Insecta	Psocodea	Caeciliusidae	*Valenzuelaflavidus* (Stephens, 1836)	nat	5	14	713	74
Insecta	Psocodea	Ectopsocidae	*Ectopsocusbriggsi* McLachlan, 1899	intr	1	23	298	15
Insecta	Psocodea	Ectopsocidae	*Ectopsocusstrauchi* Enderlein, 1906	nat		9	23	25
Insecta	Psocodea	Elipsocidae	*Elipsocusazoricus* Meinander, 1975	end		5	523	107
Insecta	Psocodea	Elipsocidae	*Elipsocusbrincki* Badonnel, 1963	end		12	627	1
Insecta	Psocodea	Epipsocidae	*Bertkauialucifuga* (Rambur, 1842)	nat			23	9
Insecta	Psocodea	Psocidae	*Atlantopsocusadustus* (Hagen, 1865)	nat		3	74	51
Insecta	Psocodea	Trichopsocidae	*Trichopsocusclarus* (Banks, 1908)	nat	8	56	618	143
Insecta	Thysanoptera	Aeolothripidae	*Aeolothripsgloriosus* Bagnall, 1914	nat	**1**			
Insecta	Thysanoptera	Phlaeothripidae	*Hoplothripscorticis* (De Geer, 1773)	nat	**3**	12		
Insecta	Thysanoptera	Thripidae	*Heliothripshaemorrhoidalis* (Bouché, 1833)	intr	**8**	4		
Insecta	Thysanoptera	Thripidae	*Hercinothripsbicinctus* (Bagnall, 1919)	intr		2		
Insecta	Trichoptera	Limnephilidae	*Limnephilusatlanticus* Nybom, 1948	end		3	1	
